# The role of the oral microbiota in the causal effect of adjunctive antibiotics on clinical outcomes in stage III–IV periodontitis patients

**DOI:** 10.1186/s40168-024-01945-3

**Published:** 2024-10-26

**Authors:** Sven Kleine Bardenhorst, Daniel Hagenfeld, Johannes Matern, Karola Prior, Inga Harks, Peter Eickholz, Katrin Lorenz, Ti-Sun Kim, Thomas Kocher, Jörg Meyle, Doğan Kaner, Yvonne Jockel-Schneider, Dag Harmsen, Benjamin Ehmke

**Affiliations:** 1 Department of Periodontology and Operative Dentistry, Muenster University Hospital, Albert-Schweitzer-Campus 1, Building W30, Münster, 48149 Germany; 2 Institute of Epidemiology and Social Medicine, University of Münster, Münster, Germany; 3 Department of Periodontology, Center for Dentistry and Oral Medicine, Goethe University Frankfurt, Frankfurt, Germany; 4 Department of Periodontology, TU Dresden, Dresden, Germany; 5 Department of Conservative Dentistry, Section of Periodontology, Clinic for Oral, Dental and Maxillofacial Diseases, Heidelberg University Hospital, Heidelberg, Germany; 6 Department of Restorative Dentistry, Periodontology, Endodontology, and Preventive and Pediatric Dentistry, University Medicine Greifswald, Greifswald, Germany; 7 Department of Periodontology, University of Giessen, Giessen, Germany; 8 Departments of Periodontology and Synoptic Dentistry, Charite-Universitätsmedizin Berlin, Berlin, Germany; 9 Department of Periodontology, Dental School, Faculty of Health, University of Witten/Herdecke, Witten, Germany; 10 Department of Periodontology, University Hospital Würzburg, Würzburg, Germany

**Keywords:** Periodontitis, Oral microbiota, Dysbiosis, Antibiotic treatment, Clinical outcomes, Subgingival plaque, Normobiotic, Directed acyclic graphs, Microbial communities, Clinical attachment loss

## Abstract

**Background:**

Periodontitis, a prevalent chronic inflammatory disease, offers insights into the broader landscape of chronic inflammatory conditions. The progression and treatment outcomes of periodontitis are closely related to the oral microbiota’s composition. Adjunctive systemic Amoxicillin 500 mg and Metronidazole 400 mg, often prescribed thrice daily for 7 days to enhance periodontal therapy’s efficacy, have lasting effects on the oral microbiome. However, the precise mechanism through which the oral microbiome influences clinical outcomes in periodontitis patients remains debated. This investigation explores the pivotal role of the oral microbiome's composition in mediating the outcomes of adjunctive systemic antibiotic treatment.

**Methods:**

Subgingival plaque samples from 10 periodontally healthy and 163 periodontitis patients from a randomized clinical trial on periodontal therapy were analyzed. Patients received either adjunctive amoxicillin/metronidazole or a placebo after mechanical periodontal treatment. Microbial samples were collected at various intervals up to 26 months post-therapy. Using topic models, we identified microbial communities associated with normobiotic and dysbiotic states, validated with 86 external and 40 internal samples. Logistic regression models evaluated the association between these microbial communities and clinical periodontitis parameters. A Directed Acyclic Graph (DAG) determined the mediating role of oral microbiota in the causal path of antibiotic treatment effects on clinical outcomes.

**Results:**

We identified clear distinctions between dysbiotic and normobiotic microbial communities, differentiating healthy from periodontitis subjects. Dysbiotic states consistently associated with below median %Pocket Probing Depth ≥ 5 mm (OR = 1.26, 95% CI [1.14–1.42]) and %Bleeding on Probing (OR = 1.09, 95% CI [1.00–1.18]). Factors like microbial response to treatment, smoking, and age were predictors of clinical attachment loss progression, whereas sex and antibiotic treatment were not. Further, we showed that the oral microbial treatment response plays a crucial role in the causal effect of antibiotic treatment on clinical treatment outcomes.

**Conclusions:**

The shift towards a normobiotic subgingival microbiome, primarily induced by adjunctive antibiotics, underscores the potential for microbiome-targeted interventions to enhance therapeutic efficacy in chronic inflammatory conditions. This study reaffirms the importance of understanding the oral microbiome's role in periodontal health and paves the way for future research exploring personalized treatment strategies based on individual microbiome profiles.

Video Abstract

**Supplementary Information:**

The online version contains supplementary material available at 10.1186/s40168-024-01945-3.

## Introduction

Bacteria play a crucial role in the maintenance of human health as well as the development of a wide spectrum of diseases, spanning neurologic, psychiatric, respiratory, cardiovascular, gastrointestinal, hepatic, autoimmune, metabolic, and oncologic conditions [31]. Within the oral biofilm, which encompasses all parts of the oral cavity, several genera have been identified as components of the core healthy human microbiome, including *Streptococcus*, *Fusobacterium*, *Prevotella*, *Rothia*, and *Neisseria* [11, 47]. However, when periodontitis occurs, there is a significant shift in the subgingival microbiota, leading to dysbiosis [27]. This dysbiotic state is characterized by an increase in overall bacterial diversity and the dominance of disease-associated pathobionts, such as *Porphyromonas*, *Fretibacteria*, *Treponema*, and *Tanerella* [1]. To quantify this dysbiosis, novel subgingival dysbiosis indices have been developed, utilizing some of those and other discriminating genera and species [12, 36].

However, it is important to acknowledge that the current microbiome indices utilized in periodontal research have certain limitations. These indices focus on specific genera or species within the microbial community, providing a limited representation of the overall microbiome composition. This approach may overlook the complex interactions and synergistic effects that occur within the entire subgingival ecosystem. Moreover, microbiome indices may fail to capture the dynamic nature of the oral microbiome and the potential contributions of less abundant taxa that could still play significant roles. To overcome these limitations, this study will employ topic models, which consider the entire microbiome composition and its inherent complexity [7, 22, 42]. By utilizing topic models, we can gain a more comprehensive and holistic understanding of the microbial clusters and their relevance to periodontal treatment outcomes.

Our previous study showed that periodontal therapy, particularly when combined with adjunctive antibiotic use, can induce long-term reductions in dysbiosis in both non-smoking and smoking periodontitis patients [24]. These reductions are achieved by decreasing the abundance of periodontal pathobionts and increasing the prevalence of commensal bacteria within the subgingival biofilm. These findings support earlier research that demonstrated short-term changes [6, 21, 37] and long-term dynamics over 1 year [6].

While the effects of mechanical periodontal treatment on clinical parameters of periodontitis have been extensively investigated [5, 8, 15, 16, 17, 28, 29, 35], the interplay between these clinical effects and the long-term dynamics of bacterial compositions in the biofilm remains unclear. Furthermore, the influence of confounding factors, such as smoking and age, on this relationship is not well understood.

The main goal of this manuscript is to investigate the role of the oral microbiota in the antibiotic treatment of periodontitis patients. This goal is achieved by (1) identifying sub-communities that characterize dysbiotic states via topic modeling, (2) identifying differential treatment responses in terms of degree dysbiosis, and (3) examining the implications of oral microbiota in the causal effect of antibiotic treatment on periodontitis treatment outcomes using a causal inference approach. By addressing these intermediate goals, we aim to elucidate the pivotal role of oral microbiota in mediating the impact of antibiotics on periodontitis treatment outcomes.

## Material and methods

### Study characteristics

This study encompassed samples from subjects with and without periodontitis, obtained from the ABPARO project (*n* = 815 samples), an internal validation (*n* = 40 samples), and external validation (Griffen study (a) *n* = 29 samples, and (b) *n* = 29 samples, and Pei study *n* = 28 samples). The periodontitis patients originated from a 26-month long-term prospective, randomized, stratified, double‐blind, multi-center (eight university hospital centers) trial with parallel‐group design about the impact of adjunctive Amoxicillin and Metronidazole on mechanical periodontal therapy (ABPARO study; 163 patients [24]. The raw reads for ABPARO samples can be publicly assessed at the European Nucleotide Archive (PRJEB51017). The healthy subjects originated from the Department of Periodontology, University Hospital Muenster, Germany (internal validation; 10 participants) Additionally, sequencing data from individuals with a healthy periodontium were obtained from the Sequence Read Archive of the National Center for Biotechnology Information [23, 39]. These datasets, utilizing various sequenced variable regions and sequencing technologies, were subjected to the same bioinformatics pipeline for consistent analysis. For further details, please refer to Table 1, which provides information on the study characteristics and accession numbers of these datasets.

**Table 1:** Datasets used for validation of identified topics

Reference	Year	Sample Size	Selection criteria	Sequencing technology	Variable region	Accession number
Hagenfeld et al.	2023	815	CAL ≥ 3mm	MiSeq (2x250bp)	V4	PRJEB51017
Griffen et al. a	2012	29	No PD >4 mm	454 GS FLX Titanium	V1-V2	SRP009299
Griffen et al. b	2012	29	No PD >4 mm	454 GS FLX Titanium	V4	SRP009299
Pei et al.	2020	28	No PD >3 mm, no CAL ≥ 1 mm	MiSeq (2x250bp)	V3-V4	SRP226726
Internal Validation	2023	40	No PD >3 mm, no periodontitis associated CAL*	MiSeq (2x250bp)	V4	N.A.

*CAL *Clinical attachment loss, *PD *pocket depth

*[38]

### Library preparation and bioinformatics

In the case of the 40 newly acquired samples of the 10 internal healthy control patients, bacterial genomic DNA was isolated and purified using a QiaAmp Mini DNA Isolation Kit (Qiagen, Hilden, Germany). The 16S—libraries were prepared, normalized, pooled, and sequenced using an Illumina MiSeq system as previously published [24]. The bioinformatics analysis utilized Illumina’s MiSeq Control Software v.2.6.2.1 and Cutadapt v.4.4 [32] for the initial processing of adapter- and primer-free FastQ files. The DADA2 package v.1.26.0 [10] in R v.4.2.2 [41] was employed for further analysis, including trimming of low-quality bases and filtering of low-quality reads as previously described [24]. Taxonomic labeling of ribosomal sequence variants (RSVs) was performed using a Bayesian classifier and the eHOMD v.15.23 [13] training set. Species assignment was done based on matching sequences with the eHOMD and SILVA v.138.1 [40] training sets, as previously described [24]. The healthy control samples obtained from [23], which were generated using 454 sequencing, underwent denoising with DADA2 using specific parameters,The maximum expected error (maxEE) threshold was adjusted to 0.2 (default is 2) to account for anticipated higher levels of spurious sequencing. No merging or prevalence filter was applied during the denoising process. Additionally, for the study conducted by [39], utilizing Illumina sequencing, we used the same denoising settings as those employed in the ABPARO-study. A minimum abundance filter of 100 was applied across all studies, to remove suspiciously rare sequence variants.

### Statistical analysis

Topic models were fitted using the *topicmodels* package v 0.2 in R to all available samples. Each identified topic represents co-occurring microbial taxa indicating communities or patterns of microbes that frequently appear together across different samples. Here, the number of topics was deliberately fixed at two to ensure easy interpretability of the identified topics as the aim was to classify topics to represent normobiotic or dysbiotic states based on the microbial contributions to each topic. By validating our topics on other healthy and untreated patients, we aimed to demonstrate that our identified topics (normobiotic and dysbiotic) are consistent reflections of a healthy oral microbiome and not merely artifacts of antibiotic targets (Table 1). The relative proportion of the dysbiotic to the normobiotic topic within each sample was used as a measure of the degree of dysbiosis. Logistic regression models were used to assess the association of degree of dysbiosis on below or above median clinical outcomes measured as a percentage of sites with pocket probing depths ≥ 5 mm (%PPD ≥ 5 mm), percentage of sites with bleeding on probing (%Bleeding), and percentage of sites with further attachment loss more than 1.3 mm compared to baseline (%AL 1.3 mm). Also, the proposed clinical endpoint of “ ≤ 4 sites with PD ≥ 5 mm” was calculated and used to link microbial composition to clinical outcomes [20]. If not stated otherwise, models were adjusted for the confounding factors age, sex, and smoking. To capture the underlying heterogeneity in microbiota response to treatment, we employed hierarchical clustering to identify distinct sub-groups that demonstrated similar patterns of microbial treatment response over time. An ordinal logistic regression model was used to assess how baseline characteristics shape microbial treatment response patterns. Effects of treatment response on %AL 1.3 mm were analyzed using negative binomial regression models. To account for variability in the total number of sites per patient, the total number of sites was included as an offset in the negative binomial regression models, effectively modeling the proportion of sites with further attachment loss.

**Fig. 1:**
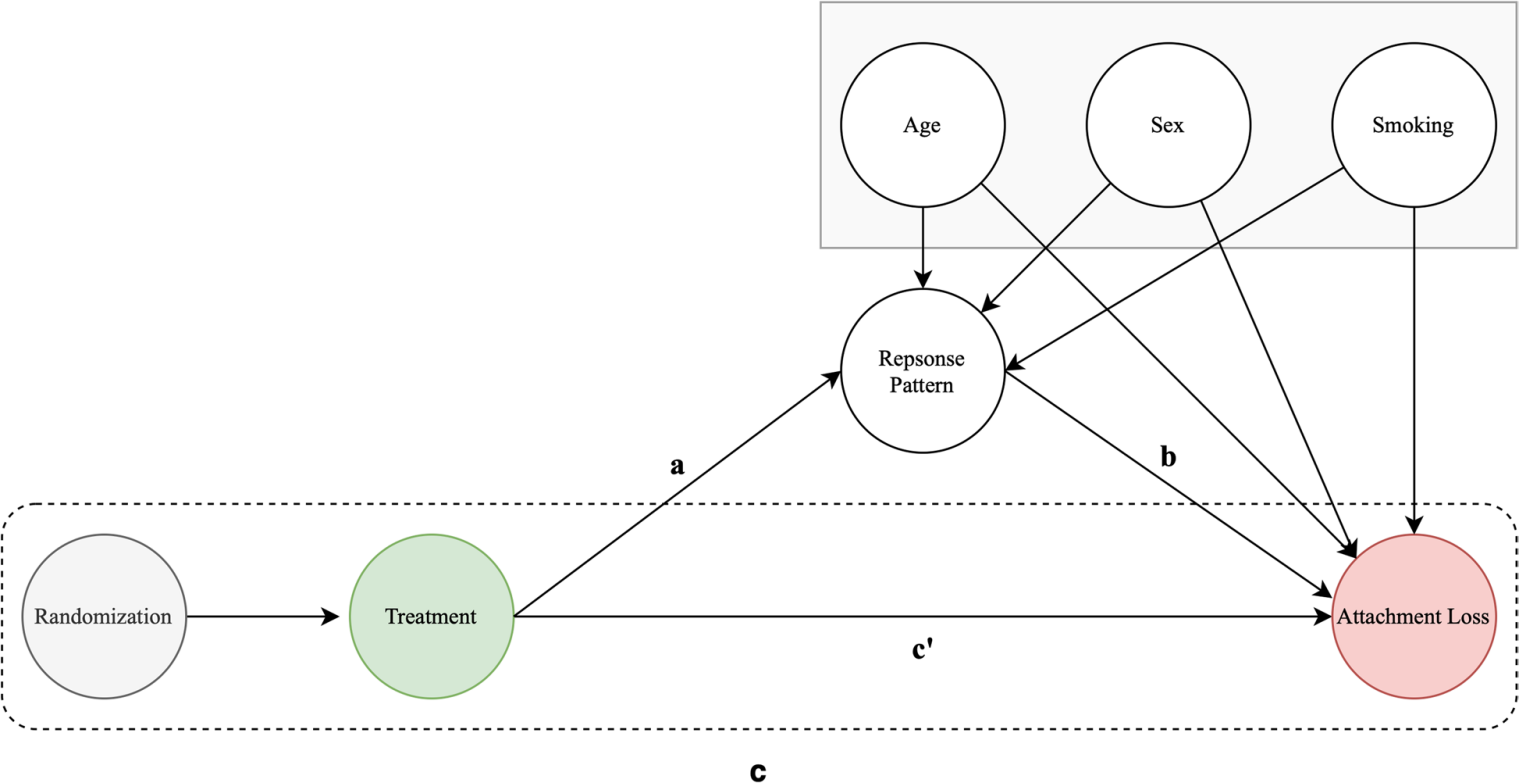
Directed acyclic graphs depicting the model approach for causal inference. Baseline characteristics influence the pattern of microbial treatment response (**a**). The effects of treatment response on %AL 1.3 mm were analyzed using negative binomial regression models (**b**). To assess the mediating effect of the response pattern, the difference method was employed. Two negative binomial models were fitted to estimate the total effect (**c**) and the direct effect (c') of antibiotic treatment on %AL 1.3 mm

To elucidate the role of the microbiome in the causal effect of antibiotic treatment on further attachment loss, we applied the principles of causal inference and causal mediation methodology [46]. We constructed a Directed Acyclic Graph (DAG) to illustrate the causal structure of these relationships (Fig. 1). The DAG allowed us to visually represent the causal structure and to identify potential confounders, therefore informing our statistical models’ specification by identifying adjustment sets. It is important to note that the DAG is specifically tailored to represent the average causal treatment effect (that is, the total effect) and the controlled direct effect (that is, the direct effect, controlled for the microbiome as potential mediator and potential confounders of the mediator-outcome association) of antibiotics on further attachment loss and should be interpreted only in this context. Two negative binomial models were fitted to estimate the total (c) and direct (c’) effect of antibiotic treatment on %AL 1.3 mm. Using Bayesian models, we obtained posterior draws of the difference in coefficients between the total and direct effects (c–c’), thereby obtaining a point estimate and a credibility interval for the indirect effect. This methodology provided a robust approach to infer the magnitude and uncertainty of the indirect effect mediated through changes in oral microbiota post-treatment. All Bayesian models were estimated using the package brms 2.18.0 [9]. DAGs and their according adjustment sets were derived using the package dagitty 0.3 [44].

## Results

### Microbial composition and dysbiosis

The application of topic models allowed us to determine the relative abundance of each topic within the oral microbiota samples, providing a measure of dysbiosis. Our results demonstrated a strong correlation (*r* = 0.81, *p* < 0.001) with a previously published Subgingival Microbial Dysbiosis Index [12] and exhibited nearly identical long-term patterns as in our previous study (Supplementary Fig. S1, [24]).

**Fig. 2:**
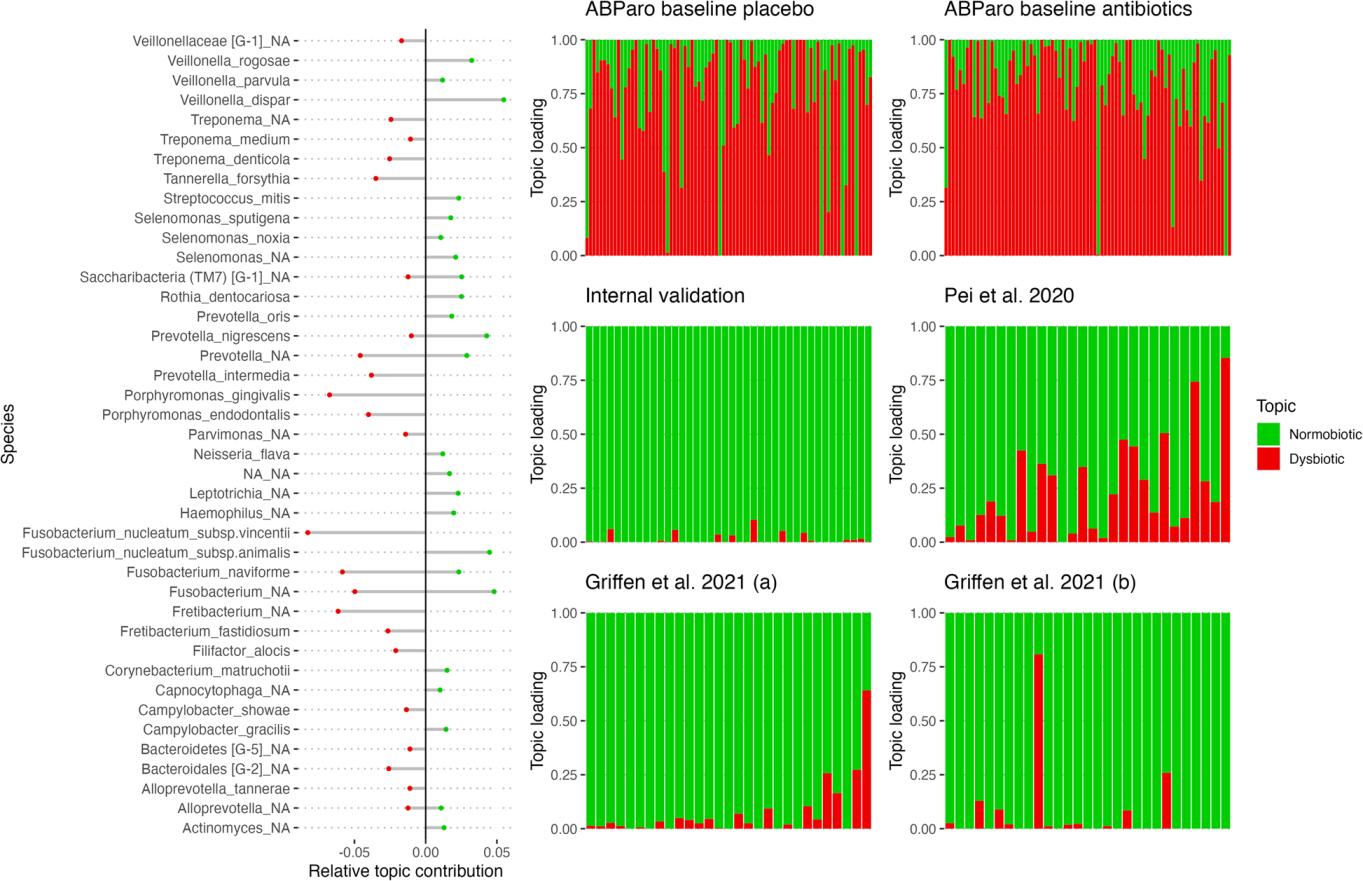
Contributions of taxa to dysbiotic (red) and normobiotic (green) topics, and topic loadings of included studies. Left side displays taxa with higher contributions to dysbiotic or normobiotic topics. Right side shows topic loadings for each of the 163 periodontitis patients, as well as healthy internal and external controls without periodontitis. [23, 39]

We identified two distinct microbial topics that effectively captured known pathogenic drivers of periodontitis (dysbiotic topic) and species commonly associated with healthy oral microbiota (normobiotic topic) (Fig. 2). The normobiotic topic was characterized by the presence of three *Veillonella* species (*rogosae*, *parvula*, and *dispar*), as well as commensal species such as *Rothia dentocariosa*, *Rothia nigrescens*, and the *animalis* subspecies of *Fusobacterium nucleatum*. Samples from healthy periodontal specimens consistently exhibited high normobiotic topic loadings, irrespective of differences in sequencing technology, hypervariable region, and study population. All studies with specimens from a healthy periodontium demonstrated significantly higher normobiotic topic loadings compared to the dysbiotic untreated periodontitis sample. The main drivers of the dysbiotic topic were the species *Porphyromonas gingivalis*, *Porphyromonas endodontalis*, *Fusobacterium nucleatum subspecies vincentii*, *Treponema denticola*, and two unclassified species from the genera *Fretibacterium* and *Prevotella* (Fig. 2).

### Treatment effects on dysbiosis

In untreated periodontitis patients, the baseline microbiome was dominated by the dysbiotic community in both the antibiotic and placebo treatment groups, with a median relative abundance of 86.02% and 87.45%, respectively. Two months after treatment, the relative abundance of the dysbiotic community significantly decreased to 2.52% in the antibiotic treatment group and 62.21% in the placebo group. Over time, the dysbiotic community increased again but remained below baseline levels for the antibiotic treatment group, with a median topic relative abundance of 48.86% even after 26 months.

### Association with clinical outcomes

The ratio of the dysbiotic community in the oral microbiome was consistently associated with worse clinical characteristics, i.e., above-median values, at each time point. Before treatment, a higher proportion of the normobaric community was strongly associated with beneficial clinical characteristics that are below the median %PPD ≥ 5 mm (OR = 1.26, 95% CI [1.14–1.42]) and %Bleeding (OR = 1.09, 95% CI [1.00–1.18]). Two months after treatment, this association persisted even after adjusting for sex, age, and antibiotic treatment. Although the strength of these associations slightly decreased over the course of follow-up, they increased again after 26 months at the end of follow-up for both %PPD ≥ 5 mm (OR = 1.14, 95% CI [1.04–1.27]) and %Bleeding (OR = 1.08, 95% CI [1.00–1.19]). Dysbiosis was associated with %CAL ≥ 1.3 mm only at 26 months after treatment (OR = 1.11, 95% CI [1.02–1.22]). Furthermore, we calculated the Treat-to-target endpoint as ≤ 4 sites with PPD ≥ 5 mm [20]. Normobiosis in the oral microbiome was associated with improved treat-to-target endpoint at 26 months after treatment (OR = 1.20, 95% CI [1.08–1.37]), but not earlier.

### Clustering to detect microbial treatment response pattern

**Fig. 3:**
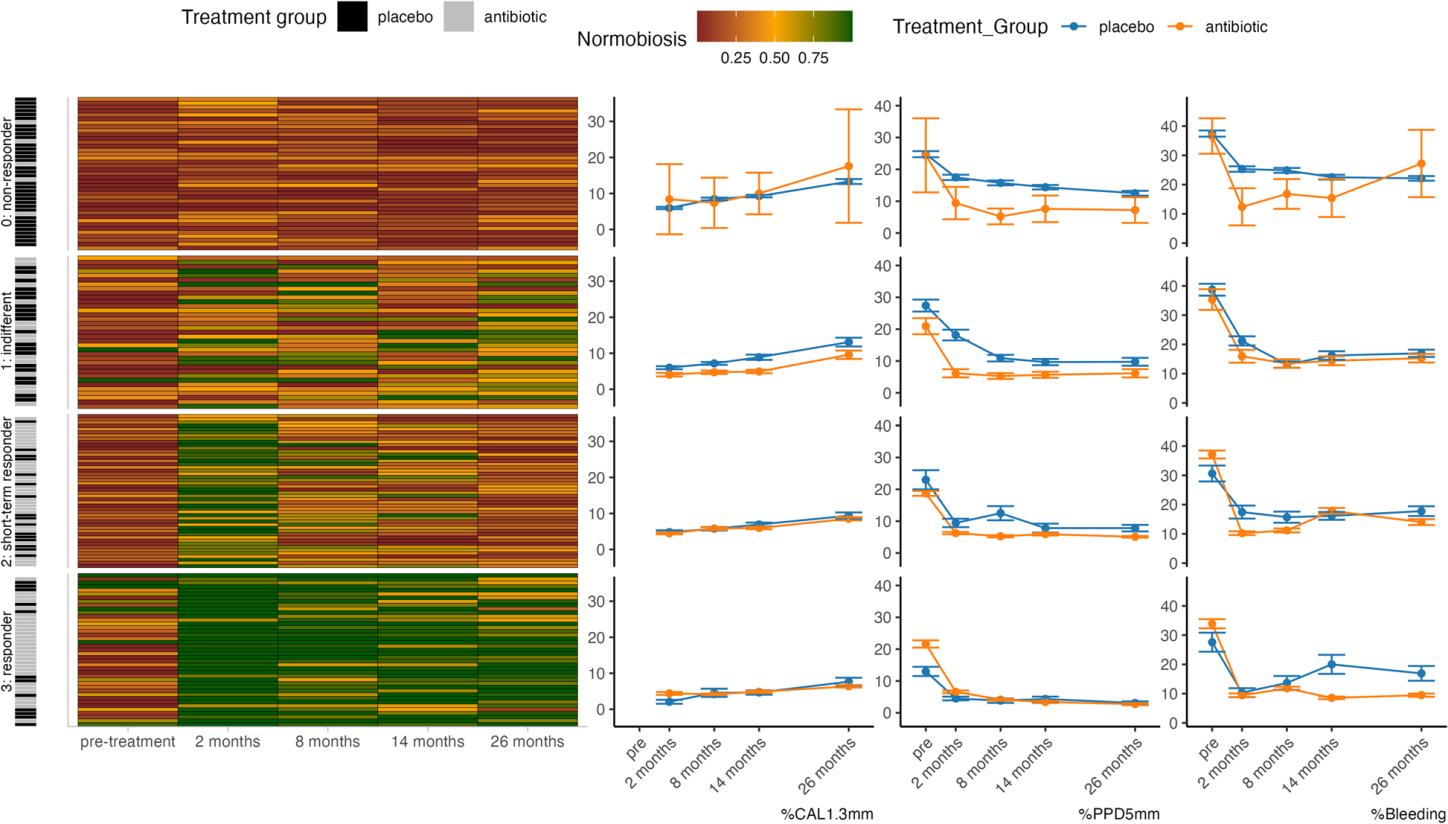
Heatmap of microbial treatment response from individual patients and line-graphs of median clinical parameters per response group over 26 months of periodontal therapy. Each row represents an individual patient summarized by the microbial treatment response: “0: non-responders” that exhibited a dysbiotic microbiome before treatment, which remained dysbiotic throughout the study. The “1: indifferent” group showed no discernible pattern in microbial dynamics. “2: short-term responders” initially transitioned to a normobiotic state after treatment but later reverted to a dysbiotic state. “3: responders” successfully transitioned to a normobiotic microbiome and maintained this state until the end of the study. % Bleeding: % Bleeding on probing; %PPD5mm: % of site showing PPD ≥ 5 mm; %AL1.3 mm: Percentage of sites with new attachment loss ≥ 1.3 mm

To further elucidate the interplay between the oral microbiota, clinical parameters, and treatment response at the individual patient level, we performed hierarchical clustering. Patients who exhibited microbial dynamics without a discernible pattern were classified as “indifferent”. Three additional subgroups with distinct microbial dynamics were identified based on their response to periodontal therapy. “Non-responders” had a dysbiotic microbiome before treatment, which did not achieve a transition to a normobiotic state after treatment. “Short-term responders” initially changed to a normobiotic microbiome after treatment but subsequently reverted to a dysbiotic state at the end of the study. Finally, “Responders” transitioned to a normobiotic microbiome and maintained this state until the end of the study (Fig. 3).

### Effects of microbial treatment response on clinical outcomes

We assume that the responder categories form an ordinal scale, premised on the observation that clinical attachment loss incrementally decreases as the microbial response category improves (hence, the order of the response categories is non-responder (lowest), indifferent, short-term responder and responder (highest)).

The ordinal logistic regression examining the effect of treatment and baseline microbiome on the microbial response pattern showed a strong association between the treatment and the microbial response pattern. Specifically, individuals who received adjunctive antibiotics (the treatment) were more likely to fall into a higher response category. The odds ratio was 6.42 (95% CI: 3.30–12.89), indicating that those in the antibiotic treatment group had approximately 6.42 times higher odds of having a higher treatment response category than those in the placebo group. Likewise, having a baseline normobiotic microbiome was associated with 7.8 (95% CI 3.79–16.05) higher odds of falling into a higher response category. A negative binomial regression model was fitted to estimate the effect of the response pattern on further attachment loss after treatment, controlling for the effects of antibiotic treatment. Here, the rate ratio was found to be 0.72 (95% CI 0.54–0.95), implying that for each increase in the treatment response category, the rate of attachment loss was reduced by approximately 28%.

### Impact of microbial treatment response on antibiotic treatment efficacy

We constructed a DAG to represent the framework of causal relationships and to identify the role of the microbial response categories and potential confounders. As antibiotic treatment was subject to randomization, we can assume that the effects of treatment are independent of any confounders, assuming that no selection bias is present. However, it is safe to assume that the set of confounders, sex, age, and smoking, will affect both, the microbial treatment response as well the clinical outcome %AL 1.3 mm. As the variables have different scales (ordinal mediator; count outcome), the most straightforward and interpretable approach to establish a mediation effect is to estimate the total and direct effect separately. A negative binomial regression was used to assess the total effect of treatment on attachment loss. Note, that this model was, as elucidated by the DAG, not adjusted for confounders. Here, the rate ratio was 0.73 (95% CI 0.57–0.93), indicating that antibiotic treatment was associated with a 27% reduction in the rate of attachment loss. We further estimated the direct effect of treatment on attachment loss (model c’), by adjusting for the response pattern along with age, sex, and smoking. The rate ratio for the direct effect of treatment was found to be 0.82 (95% CI 0.62–1.07), suggesting that after adjusting for the mediator and confounders of the mediator-outcome association, there was no more evidence for the antibiotic treatment effect on attachment loss. This change in the antibiotic treatment effect, when accounting for the response pattern, provides evidence for the mediating role of the microbial response pattern in the relationship between adjunctive antibiotics and attachment loss. However, to further investigate this mediation, we used the posterior draws of the estimates of the total and direct (c’) effect and calculated the indirect effect (c–c’). The indirect effect was 0.89 (95% CI 0.62–1.31), indicating high variance in the mediating effect. Therefore, while there was no evidence for a direct effect after adjusting for the mediator, the high variance in the indirect effect implies that we cannot conclusively assert the presence of mediation.

### Predicted further sites with attachment loss

Using the microbial response categories, we obtained a predicted number of sites with further attachment loss, considering the influence of antibiotic treatment and placebo across diverse age groups and smoking statuses. The data is presented as model predictions, representing the hypothetical absolute number of sites with new attachment loss ≥ 1.3 mm over a 26-month periodontal therapy for a patient with 24 teeth who has a total of 126 sites measured (Table 2). By comparing these numbers, we can assess the relative contributions of each individual effect on the absolute numbers of sites with further attachment loss ≥ 1.3 mm, while all other effects are constant.

**Table 2:** Expected number of sites with further attachment loss of more than 1.3 mm, 26 months after mechanical periodontal therapy, based on model predictions. The table presents expected treatment outcomes based on microbial response patterns to antibiotic treatment and placebo across distinct age groups and smoking statuses. All values are expected outcomes for a patient with the number of sites measured being 146 (~24 teeth, the average number of teeth in this study)

		Antibiotic				Placebo			
Smoking	Age	Non-responder	Indifferent	Short-term responder	Long-term responder	Non-responder	Indifferent	Short-term responder	Long-term responder
Non-smoker	< 45	6.55	6.02	4.72	4.47	8.14	7.46	5.84	5.56
Non-smoker	45 < 55	7.74	7.11	5.56	5.27	9.60	8.77	6.89	6.54
Non-smoker	> 55	9.60	8.82	6.93	6.93	11.93	10.96	8.58	8.16
Smoker	< 45	12.06	11.05	8.69	8.69	14.91	13.70	10.77	10.23
Smoker	45 < 55	14.22	13.04	10.23	10.23	17.63	16.13	12.69	12.09
Smoker	> 55	17.67	16.62	12.74	12.74	21.95	20.11	15.78	15.02

To illustrate this approach, one may consider a hypothetical non-smoker with 8 sites experiencing further attachment loss ≥ 1.3 mm. The model estimates a 19% reduction in attachment loss with antibiotics, leading to only 6.5 sites with attachment loss if this patient would have been treated with antibiotics. However, note, that the effects of the model are multiplicative. Say, the same individual is a smoker, the model would assume an 85% increase in the number of sites with attachment loss, resulting in 14.91 sites. Thus, the 19% decrease due to antibiotics now corresponds to a decrease of 2.8 sites. Importantly, the effect of a better microbial treatment response is stronger than the additional effect of the antibiotic treatment, supporting our previous findings of a mediating role of microbial treatment response in adjunctive antibiotic use. This comparative analysis allows for a deeper understanding of how various factors influence the outcomes of periodontal therapy with or without adjunctive antibiotics and emphasizes the importance of individualized treatment considerations.

## Discussion

In our study, we undertook a robust approach to understand the role of the oral microbiome in the relationship between antibiotic treatment and periodontal health, adopting methodology from the causal inference framework. Key findings from our analysis reveal the substantial impact of the oral microbiome on periodontal health, highlighting the importance of individual microbial profiles in treatment outcomes. We further highlight how baseline characteristics shape the individual treatment response and how substantial parts of the effect of antibiotic treatment on periodontal health might be mediated through individual responses in the oral microbiome of patients.

The identification of two distinct microbial topics, the dysbiotic and normobiotic, allowed us to reliably capture the health status of the oral microbiota of patients. The dysbiotic topic predominantly consisted of well-known periodontal pathogens such as *Porphyromonas gingivalis*, *Porphyromonas endodontalis*, *Treponema denticola*, and *Fusobacterium nucleatum* [2], while the normobiotic topic included species commonly associated with a healthy oral microbiota such as *Veillonella* and *Rothia* species [47]. These findings support the notion that dysbiosis in the oral microbiota involves a shift towards pathogenic microbial communities, accompanied by a reduction in beneficial commensal species [26]. A strong correlation between our topic model-based dysbiosis measure (Supplementary Fig. S1) and a previously established Subgingival-Microbial-Dysbiosis-Index [12], further indicated the reliability and validity of our approach. However, our method provided a comprehensive view of dysbiosis by considering the relative contributions of multiple microbial topics, rather than focusing on a limited set of pathogenic bacteria. We further showed the stability and generalizability of the topics across different study populations and methodologies [23, 39]. Regardless of differences in sequencing technology, hypervariable regions, and study populations, healthy periodontal specimens consistently exhibited high normobiotic topic relative abundance. This indicates that the normobiotic topic represents a robust indicator of a healthy oral microbiota. The identification of a dominant normobiotic topic in healthy subjects further emphasizes its potential as a useful reference for assessing dysbiosis in periodontitis patients.

Assessing the impact of periodontal treatment on dysbiosis, we found that both antibiotic and placebo treatment groups initially exhibited high relative abundances of the dysbiotic topic (Fig. 2). However, two months after treatment, the dysbiotic topic significantly decreased in the antibiotic group, indicating the effectiveness of antibiotic intervention in reducing the pathogenic microbial community [24]. In contrast, the placebo group showed a partial reduction in the dysbiotic topic but remained at relatively high levels of the dysbiotic topic relative abundance. These findings suggest that antibiotic treatment had a more profound and sustained effect on dysbiosis compared to placebo, highlighting the potential therapeutic value of targeted microbial interventions [34, 43].

However, whether a patient benefits from periodontal therapy depends on the change in the microbiome after therapy and not on whether the change in the microbiome was achieved by adjunct antibiotics or placebo. In addition, smoking status proved to be informative for the potential clinical response to treatment. According to our model (Table 2), non-smoking patients (< 45 years) with the microbiome pattern “long-term responder” will have an absolute number of attachment losses at 5.42 sites after adjunct antibiotics or at 6.63 sites with placebo. In smokers, on the other hand, the number of sites with further attachment loss will be 10.03 and 12.25 sites respectively. Our results therefore suggest that it would make more sense for patients to stop smoking than to be prescribed antibiotics.

The association between dysbiosis and clinical outcomes revealed consistent patterns throughout the study period. Higher proportions of the dysbiotic topic were consistently associated with worse clinical outcomes, including increased probing depth and bleeding. Conversely, a higher proportion of the normobiotic topic was associated with beneficial clinical outcomes, including lower %PPD ≥ 5 mm and reduced percentage of bleeding. These associations remained significant even after adjusting for potential confounding factors, indicating the independent contribution of dysbiosis to the severity of periodontal disease. Notably, the association between dysbiosis and clinical attachment loss remained significant after 26 months, suggesting the long-term impact of dysbiotic microbial communities on periodontal tissue destruction supporting pathobiont-based models for the prediction of periodontal destruction [45].

The classification of the microbial treatment response allowed us to group patients into distinct subgroups based on their microbial response to treatment. The “indifferent” subgroup exhibited microbial fluctuations without a clear pattern, indicating the lack of treatment influence on their oral microbiota. Conversely, the “non-responder” subgroup maintained a dysbiotic microbiome throughout the study, suggesting their resistance to treatment-induced changes. The “short-term responder” subgroup initially responded to treatment by transitioning to a normobiotic state but subsequently reverted to a dysbiotic state, indicating a reduced treatment efficacy. Finally, the “responder” subgroup demonstrated a sustained transition to a normobiotic microbiome, highlighting their favorable treatment response by transitioning into a microbial state comparable to that of healthy patients.

To disentangle the role of the oral microbiota in the mechanisms involved in the effect of antibiotic treatment on attachment loss we investigate each potential pathway separately. The results of the ordinal logistic regression analysis revealed that baseline characteristics are predictive of differential treatment response and highlight the role of baseline information in periodontal therapy, especially the baseline microbiome, as previously shown by the group of Zaura and others [6]. The analysis of the second path revealed that better treatment response in the oral microbiota is highly associated with less clinical attachment loss. Those results give evidence that the effect of antibiotic treatment on clinical attachment loss might be mediated through response to treatment in the oral microbiota. We borrowed approaches from mediation methodology to further investigate the mediating role of the oral microbiome and the difference in the total and direct causal effect of antibiotic treatment on attachment loss further supports this finding. However, the estimate of the magnitude of the indirect effect (the difference between the total and the direct effect) shows high variability and should be interpreted with care, as the analysis was designed to establish the presence, not the magnitude of the mediating effect. Therefore, the analysis did not allow for an unbiased estimation of the causal mediation effect, as not all assumptions could be met. Specifically, there might be more factors that confound the association of the microbiome and the outcome that could not be controlled for in this study, i.e., compliance to oral hygiene, amount of smoking, prediabetes, or nutrition.

In a previous descriptive exploratory subgroup analysis of the ABPARO trial [16], age and %PPD ≥ 5 mm were suggested to influence periodontitis progression. Our findings confirm the relevant role of age. However, due to the nature of our modeling approach, we cannot directly control for %PPD ≥ 5 mm, as this variable is directly linked to our outcome. Despite this limitation, our model-based approach proves to be more robust against outliers compared to the exploratory analyses conducted in the previous study. Furthermore, the effect of the treatment, as well as the association with treatment response might be moderated by other factors (i.e., disease progression). This assumption is also supported by the recent finding that the clinical benefit of antibiotic treatment is dependent on disease progression [15]. We can confirm similar findings on the total effect of antibiotic treatment in our study (Supplementary Table S1), however, we could not investigate this behavior in detail, as no information on the periodontitis grade was available for 46.6% of the participants. This finding underscores the importance of microbial shifts as a key mechanism underlying the efficacy of antibiotic treatment in periodontal therapy [14, 21, 34, 37]. Specifically, if antibiotics fail to induce the desired alterations in the oral microbiome, their impact on attachment loss may be limited. This highlights the need to consider individual microbial profiles and responses when determining the most effective treatment strategies [3, 4].

Furthermore, smoking and the categories of short- and long-term responses emerged as significant predictors of treatment outcomes. The influence of smoking on periodontal health and treatment response has been extensively documented, and our findings align with previous research indicating that smoking negatively affects treatment outcomes [18, 19, 25, 30, 33]. The identification of short- and long-term response categories as significant predictors suggests the existence of distinct subgroups with varying treatment responses, which may warrant tailored therapeutic approaches.

Our study introduces a novel approach to topic identification that shares similarities with the results obtained through SMDI but offers greater flexibility and generalizability. Unlike SMDI, which focuses on specific dysbiotic patterns, our method considers the entirety of the microbiome and enables us to address more complex research questions. Furthermore, the inclusion of additional topics allows for the identification of sub-topics that are vital for examining fine-scale associations and making individualized predictions. To enhance the robustness of our analysis, future studies could consider refining the analytical approach by potentially incorporating a structural equation model. This advanced modeling technique would enable a comprehensive evaluation of the interrelationships among baseline factors, treatment response, and attachment loss, thereby offering a more comprehensive understanding of the underlying dynamics. It is worth noting that the taxonomic resolution achieved in our study was limited to the genus level due to the utilization of short reads. While this level of resolution provided valuable insights, future investigations employing long-read sequencing technologies could potentially offer a higher taxonomic resolution, enabling a more detailed characterization of the oral microbiota.

Our findings strongly endorse the concept that dysbiosis, marked by the prevalence of pathogenic microbial communities, plays a pivotal role in the degradation of periodontal tissues. The identification of a normobiotic topic linked with a healthy oral condition underscores its potential as a valuable biomarker and therapeutic target in the context of periodontal management. Beyond this, our research hints at a broader implication—the potential use of oral microbial biomarker screening as a readily accessible tool to monitor the course of periodontal disease. As we move forward, we must emphasize the need for further research exploring the ripple effects of dysbiotic oral microbiota on systemic conditions. Such investigations have the potential to uncover novel links between periodontal and systemic health, offering valuable insights into personalized treatment approaches. This avenue of research holds promise not only for periodontal care but also for enhancing our understanding of systemic health in a broader context.

## Conclusion

In conclusion, this study provides important insights into the predictability of responder categories based on baseline information and sheds light on the critical role of the oral microbiome in mediating the effects of antibiotics on attachment loss. These findings highlight the need for personalized treatment approaches that consider individual microbial profiles and indicate the significance of smoking and the categorization of treatment response in influencing treatment outcomes. Further research, including refined analytical approaches, can contribute to a deeper understanding of the complex interplay between baseline factors, treatment response, and clinical periodontal outcomes.

## Supplementary Information

Supplementary Material 1.

## Data Availability

The raw sequencing data used and/or analysed during the current study are available via the ENA as stated in the manuscript.  Patient information cannot be published without patients consent. Please contact the corresponding author for reasonable request.

## Abbreviations

DAG: Directed Acyclic Graph
OR: Odds ratio
CI: Confidence Interval
ABPARO –Trial: Multicenter randomized controlled study about the additional long-term benefit of adjunctive antibiotics on periodontal treatment

## References

[1] Abusleme L, Hoare A, Hong B-Y, Diaz PI. Microbial signatures of health, gingivitis, and periodontitis. Periodontology 2000. 2021;86(1):57–78. 10.1111/prd.12362. 33690899 10.1111/prd.12362

[2] Àlvarez G, Arredondo A, Isabal S, Teughels W, Laleman I, Contreras MJ, Isbej L, Huapaya E, Mendoza G, Mor C, Nart J, Blanc V, León R. Association of nine pathobionts with periodontitis in four South American and European countries. J Oral Microbiol. 2023;15(1):2188630. 10.1080/20002297.2023.2188630. 36950255 10.1080/20002297.2023.2188630 PMC10026778

[3] Belibasakis, G. N., Belstrøm, D., Eick, S., Gursoy, U. K., Johansson, A., & Könönen, E. (2023). Periodontal microbiology and microbial etiology of periodontal diseases: historical concepts and contemporary perspectives. Periodontology 2000, prd.12473. 10.1111/prd.12473 10.1111/prd.12473 36661184

[4] Belibasakis GN, Bostanci N, Marsh PD, Zaura E. Applications of the oral microbiome in personalized dentistry. Arch Oral Biol. 2019;104:7–12. 10.1016/j.archoralbio.2019.05.023. 31153099 10.1016/j.archoralbio.2019.05.023

[5] Berglundh T, Krok L, Liljenberg B, Westfelt E, Serino G, Lindhe J. The use of metronidazole and amoxicillin in the treatment of advanced periodontal disease. J Clin Periodontol. 1998;25(5):354–62. 10.1111/j.1600-051X.1998.tb02455.x. 9650870 10.1111/j.1600-051x.1998.tb02455.x

[6] Bizzarro S, Laine ML, Buijs MJ, Brandt BW, Crielaard W, Loos BG, Zaura E. Microbial profiles at baseline and not the use of antibiotics determine the clinical outcome of the treatment of chronic periodontitis. Sci Rep. 2016;6(1):20205. 10.1038/srep20205. 26830979 10.1038/srep20205 PMC4735321

[7] Blei DM. Probabilistic topic models. Commun ACM. 2012;55(4):77–84. 10.1145/2133806.2133826.

[8] Borges I, Faveri M, Figueiredo LC, Duarte PM, Retamal-Valdes B, Montenegro SCL, Feres M. Different antibiotic protocols in the treatment of severe chronic periodontitis: a 1-year randomized trial. J Clin Periodontol. 2017;44(8):822–32. 10.1111/jcpe.12721. 28303587 10.1111/jcpe.12721

[9] Bürkner, P.-C. (2017). brms: an *R* package for Bayesian multilevel models using *Stan*. *Journal of Statistical Software*, *80*(1). 10.18637/jss.v080.i01

[10] Callahan BJ, McMurdie PJ, Rosen MJ, Han AW, Johnson AJA, Holmes SP. DADA2: High-resolution sample inference from Illumina amplicon data. Nat Methods. 2016;13(7):581–3. 10.1038/nmeth.3869. 27214047 10.1038/nmeth.3869 PMC4927377

[11] Caporaso JG, Lauber CL, Costello EK, Berg-Lyons D, Gonzalez A, Stombaugh J, Knights D, Gajer P, Ravel J, Fierer N, Gordon JI, Knight R. Moving pictures of the human microbiome. Genome Biol. 2011;12(5):R50. 10.1186/gb-2011-12-5-r50. 21624126 10.1186/gb-2011-12-5-r50 PMC3271711

[12] Chen T, Marsh PD, Al-Hebshi NN. SMDI: an index for measuring subgingival microbial dysbiosis. J Dent Res. 2022;101(3):331–8. 10.1177/00220345211035775. 34428955 10.1177/00220345211035775 PMC8982011

[13] Chen, T., Yu, W.-H., Izard, J., Baranova, O. V., Lakshmanan, A., & Dewhirst, F. E. (2010). The human oral microbiome database: a web accessible resource for investigating oral microbe taxonomic and genomic information. *Database*, *2010*(0), baq013–baq013. 10.1093/database/baq013 10.1093/database/baq013 PMC2911848 20624719

[14] Dilber E, Hagenfeld D, Ehmke B, Faggion CM. A systematic review on bacterial community changes after periodontal therapy with and without systemic antibiotics: an analysis with a wider lens. J Periodontal Res. 2020;55(6):785–800. 10.1111/jre.12803. 32990996 10.1111/jre.12803

[15] Eickholz, P., Koch, R., Göde, M., Nickles, K., Kocher, T., Lorenz, K., Kim, T., Meyle, J., Kaner, D., Schlagenhauf, U., Harks, I., & Ehmke, B. (2023). Clinical benefits of systemic amoxicillin/metronidazole may depend on periodontitis stage and grade: An exploratory sub‐analysis of the ABPARO trial. *Journal of Clinical Periodontology*, jcpe.13838. 10.1111/jcpe.13838 10.1111/jcpe.13838 37293896

[16] Eickholz P, Koch R, Kocher T, Hoffmann T, Kim T, Meyle J, Kaner D, Schlagenhauf U, Harmsen D, Harks I, Ehmke B. Clinical benefits of systemic amoxicillin/metronidazole may depend on periodontitis severity and patients’ age: an exploratory sub-analysis of the ABPARO trial. J Clin Periodontol. 2019;46(4):491–501. 10.1111/jcpe.13096. 30825384 10.1111/jcpe.13096 PMC6594242

[17] Eickholz P, Nickles K, Koch R, Harks I, Hoffmann T, Kim T, Kocher T, Meyle J, Kaner D, Schlagenhauf U, Doering S, Gravemeier M, Ehmke B. Is furcation involvement affected by adjunctive systemic amoxicillin plus metronidazole? A clinical trials exploratory subanalysis. J Clin Periodontol. 2016;43(10):839–48. 10.1111/jcpe.12594. 27393928 10.1111/jcpe.12594

[18] Faveri M, Rebello A, de Oliveira Dias R, Borges-Junior I, Duarte PM, Figueiredo LC, Feres M. Clinical and microbiologic effects of adjunctive metronidazole plus amoxicillin in the treatment of generalized chronic periodontitis: smokers versus non-smokers. J Periodontol. 2014;85(4):581–91. 10.1902/jop.2013.130278. 23826648 10.1902/jop.2013.130278

[19] Feres M, Bernal M, Matarazzo F, Faveri M, Duarte P, Figueiredo L. Subgingival bacterial recolonization after scaling and root planing in smokers with chronic periodontitis. Aust Dent J. 2015;60(2):225–32. 10.1111/adj.12225. 25283721 10.1111/adj.12225

[20] Feres M, Retamal-Valdes B, Faveri M, Duarte P, Shibli J, Soares GMS, Miranda T, Teles F, Goodson M, Hasturk H, Van Dyke T, Ehmke B, Eickholz P, Schlagenhauf U, Meyle J, Koch R, Kocher T, Hoffmann T, Kim T-S, Doyle H, et al. Proposal of a clinical endpoint for periodontal trials: the treat-to-target approach. J Int Acad Periodontol. 2020;22(2):41–53. 32224549

[21] Feres M, Retamal-Valdes B, Fermiano D, Faveri M, Figueiredo LC, Mayer MPA, Lee J, Bittinger K, Teles F. Microbiome changes in young periodontitis patients treated with adjunctive metronidazole and amoxicillin. J Periodontol. 2021;92(4):467–78. 10.1002/JPER.20-0128. 32844406 10.1002/JPER.20-0128

[22] Fukuyama, J., Sankaran, K., & Symul, L. (2021). *Multiscale analysis of count data through topic alignment*. 10.48550/ARXIV.2109.05541 10.1093/biostatistics/kxac018 35657012

[23] Griffen AL, Beall CJ, Campbell JH, Firestone ND, Kumar PS, Yang ZK, Podar M, Leys EJ. Distinct and complex bacterial profiles in human periodontitis and health revealed by 16S pyrosequencing. ISME J. 2012;6(6):1176–85. 10.1038/ismej.2011.191. 22170420 10.1038/ismej.2011.191 PMC3358035

[24] Hagenfeld, D., Kleine Bardenhorst, S., Matern, J., Prior, K., Harks, I., Eickholz, P., Lorenz, K., Kim, T., Kocher, T., Meyle, J., Kaner, D., Schlagenhauf, U., Harmsen, D., & Ehmke, B. (2023). Long‐term changes in the subgingival microbiota in patients with stage III–IV periodontitis treated by mechanical therapy and adjunctive systemic antibiotics: a secondary analysis of a randomized controlled trial. *Journal of Clinical Periodontology*, jcpe.13824. 10.1111/jcpe.13824 10.1111/jcpe.13824 37160709

[25] Hagenfeld, D., Matern, J., Prior, K., Harks, I., Eickholz, P., Lorenz, K., Kim, T.-S., Kocher, T., Meyle, J., Kaner, D., Schlagenhauf, U., Harmsen, D., & Ehmke, B. (2020). Significant short-term shifts in the microbiomes of smokers with periodontitis after periodontal therapy with amoxicillin & metronidazole as revealed by 16S rDNA amplicon next generation sequencing. *Frontiers in Cellular and Infection Microbiology*, *10*. 10.3389/fcimb.2020.00167 10.3389/fcimb.2020.00167 PMC7232543 32477961

[26] Hajishengallis, G. (2023). Illuminating the oral microbiome and its host interactions: animal models of disease. *FEMS Microbiology Reviews*, fuad018. 10.1093/femsre/fuad018 10.1093/femsre/fuad018 PMC10198557 37113021

[27] Hajishengallis, G., & Lamont, R. J. (2021). Polymicrobial communities in periodontal disease: Their quasi‐organismal nature and dialogue with the host. *Periodontology 2000*, *86*(1), 210–230. 10.1111/prd.12371 10.1111/prd.12371 PMC8957750 33690950

[28] Harks I, Harmsen D, Gravemeier M, Prior K, Koch R, Doering S, Petersilka G, Weniger T, Eickholz P, Hoffmann T, Kim T-S, Kocher T, Meyle J, Purucker P, Schlagenhauf U, Ehmke B. A concept for clinical research triggered by suggestions from systematic reviews about adjunctive antibiotics. Appl Clin Res Clin Trials Regulat Affairs. 2014;1(1):43–50. 10.2174/2213476X01666140327211914.

[29] Harks I, Koch R, Eickholz P, Hoffmann T, Kim T, Kocher T, Meyle J, Kaner D, Schlagenhauf U, Doering S, Holtfreter B, Gravemeier M, Harmsen D, Ehmke B. Is progression of periodontitis relevantly influenced by systemic antibiotics? A clinical randomized trial. J Clin Periodontol. 2015;42(9):832–42. 10.1111/jcpe.12441. 26250060 10.1111/jcpe.12441 PMC5054899

[30] Joshi V, Matthews C, Aspiras M, de Jager M, Ward M, Kumar P. Smoking decreases structural and functional resilience in the subgingival ecosystem. J Clin Periodontol. 2014;41(11):1037–47. 10.1111/jcpe.12300. 25139209 10.1111/jcpe.12300

[31] Lynch SV, Pedersen O. The human intestinal microbiome in health and disease. N Engl J Med. 2016;375(24):2369–79. 10.1056/NEJMra1600266. 27974040 10.1056/NEJMra1600266

[32] Martin, M. (2011). Cutadapt removes adapter sequences from high-throughput sequencing reads. *EMBnet.Journal*, *17*(1), 10. 10.14806/ej.17.1.200

[33] Matarazzo F, Figueiredo LC, Cruz SEB, Faveri M, Feres M. Clinical and microbiological benefits of systemic metronidazole and amoxicillin in the treatment of smokers with chronic periodontitis: a randomized placebo-controlled study. J Clin Periodontol. 2008;35(10):885–96. 10.1111/j.1600-051X.2008.01304.x. 18727657 10.1111/j.1600-051X.2008.01304.x

[34] Mendes SDNC, Esteves CM, Mendes JAV, Feres M, Figueiredo N, De Miranda TS, Shibli JA, Figueiredo LC. Systemic antibiotics and chlorhexidine associated with periodontal therapy: microbiological effect on intraoral surfaces and saliva. Antibiotics. 2023;12(5):847. 10.3390/antibiotics12050847. 37237750 10.3390/antibiotics12050847 PMC10215836

[35] Mestnik MJ, Feres M, Figueiredo LC, Soares G, Teles RP, Fermiano D, Duarte PM, Faveri M. The effects of adjunctive metronidazole plus amoxicillin in the treatment of generalized aggressive periodontitis: a 1-year double-blinded, placebo-controlled, randomized clinical trial. J Clin Periodontol. 2012;39(10):955–61. 10.1111/j.1600-051X.2012.01932.x. 22882646 10.1111/j.1600-051X.2012.01932.x

[36] Najmanova L, Sabova L, Lenartova M, Janatova T, Mysak J, Vetrovsky T, Tesinska B, Balikova Novotna G, Koberska M, Broukal Z, Duskova J, Podzimek S, Janata J. R/G value—a numeric index of individual periodontal health and oral microbiome dynamics. Front Cell Infect Microbiol. 2021;11:602643. 10.3389/fcimb.2021.602643. 33777830 10.3389/fcimb.2021.602643 PMC7988090

[37] Nibali L, Sousa V, Davrandi M, Spratt D, Alyahya Q, Dopico J, Donos N. Differences in the periodontal microbiome of successfully treated and persistent aggressive periodontitis. J Clin Periodontol. 2020;47(8):980–90. 10.1111/jcpe.13330. 32557763 10.1111/jcpe.13330

[38] Papapanou, P. N., Sanz, M., Buduneli, N., Dietrich, T., Feres, M., Fine, D. H., Flemmig, T. F., Garcia, R., Giannobile, W. V., Graziani, F., Greenwell, H., Herrera, D., Kao, R. T., Kebschull, M., Kinane, D. F., Kirkwood, K. L., Kocher, T., Kornman, K. S., Kumar, P. S., … Tonetti, M. S. (2018). Periodontitis: consensus report of workgroup 2 of the 2017 World Workshop on the Classification of Periodontal and Peri-Implant Diseases and Conditions: classification and case definitions for periodontitis. *Journal of Clinical Periodontology*, *45*, S162–S170. 10.1111/jcpe.12946 10.1111/jcpe.12946 29926490

[39] Pei J, Li F, Xie Y, Liu J, Yu T, Feng X. Microbial and metabolomic analysis of gingival crevicular fluid in general chronic periodontitis patients: Lessons for a predictive, preventive, and personalized medical approach. EPMA J. 2020;11(2):197–215. 10.1007/s13167-020-00202-5. 32547651 10.1007/s13167-020-00202-5 PMC7272536

[40] Pruesse E, Quast C, Knittel K, Fuchs BM, Ludwig W, Peplies J, Glockner FO. SILVA: a comprehensive online resource for quality checked and aligned ribosomal RNA sequence data compatible with ARB. Nucleic Acids Res. 2007;35(21):7188–96. 10.1093/nar/gkm864. 17947321 10.1093/nar/gkm864 PMC2175337

[41] RStudio Team. RStudio: Integrated Development Environment for R. Boston: RStudio, Inc.; 2019. Available from: http://www.rstudio.com/.

[42] Symul L, Jeganathan P, Costello EK, France M, Bloom SM, Kwon DS, Ravel J, Relman DA, Holmes S. *Sub-communities of the vaginal ecosystem in pregnant and non-pregnant women*. Microbiology. 2021. 10.1101/2021.12.10.471327. 10.1098/rspb.2023.1461 PMC10685114 38018105

[43] Teughels W, Feres M, Oud V, Martín C, Matesanz P, Herrera D. Adjunctive effect of systemic antimicrobials in periodontitis therapy: a systematic review and meta-analysis. J Clin Periodontol. 2020;47(S22):257–81. 10.1111/jcpe.13264. 31994207 10.1111/jcpe.13264

[44] Textor, J., Van Der Zander, B., Gilthorpe, M. S., Liśkiewicz, M., & Ellison, G. T. H. (2017). Robust causal inference using directed acyclic graphs: The R package ‘dagitty.’ *International Journal of Epidemiology*, dyw341. 10.1093/ije/dyw341 10.1093/ije/dyw341 28089956

[45] Tomás I, Regueira-Iglesias A, López M, Arias-Bujanda N, Novoa L, Balsa-Castro C, Tomás M. Quantification by qPCR of pathobionts in chronic periodontitis: development of predictive models of disease severity at site-specific level. Front Microbiol. 2017;8:1443. 10.3389/fmicb.2017.01443. 28848499 10.3389/fmicb.2017.01443 PMC5552702

[46] VanderWeele TJ. Mediation analysis: a practitioner’s guide. Annu Rev Public Health. 2016;37(1):17–32. 10.1146/annurev-publhealth-032315-021402. 26653405 10.1146/annurev-publhealth-032315-021402

[47] Zaura E, Keijser BJ, Huse SM, Crielaard W. Defining the healthy “core microbiome” of oral microbial communities. BMC Microbiol. 2009;9(1):259. 10.1186/1471-2180-9-259. 20003481 10.1186/1471-2180-9-259 PMC2805672

