# Supplementary material for: The role of the oral microbiota in the causal effect of adjunctive antibiotics on clinical outcomes in stage III–IV periodontitis patients
Source: Microbiome. 2024 Oct 26;12:220. doi: 10.1186/s40168-024-01945-3 (PMC11515798; doi:10.1186/s40168-024-01945-3)
Supplement: Supplementary file 2 — Supplementary Material 1. [file 40168_2024_1945_MOESM1_ESM.zip › analysis_workflow.pdf]

Analysis Workflow

Dr. rer. medic. Sven Kleine Bardenhorst

2024-06-16

```
tse <- readRDS("Data/final_tse.RDS")
```

Filtering

Quality filtering was applied per run during bioinformatics processing. ASVs with prevalence below 2 and absolute abundance below 100 weere removed per run. As samples were removed due to missing data, the abundance filter is now applied again on the whole dataset, again removing all ASVs with an absolute abundance below 100.

```
n_orig <- nrow(tse)

abundance_filter <- rowSums(assays(tse)$counts) >= 100
tse <- tse[abundance_filter,]
n_new <- nrow(tse)
data.frame("Step"=c("Original number of ASVs:",
                          "ASVs filtered due to abundance <= 100:",
                          "New number of ASVs:"),
           ASVs = c(n_orig,
                    sum(abundance_filter == F),
                    n_new)) %>%
  kbl() %>%
  kable_styling(latex_options = c("striped","HOLD_position"))
```

Step	ASVs
Original number of ASVs:	2416
ASVs filtered due to abundance <= 100:	0
New number of ASVs:	2416

Topic modeling

```
tse_topic <- tse


rowData(tse_topic)[,7] <- paste0(rowData(tse_topic)[,6],"_",rowData(tse_topic)[,7])

tse_topic <- mia::agglomerateByRank(tse_topic,"Species",onRankOnly=T)
assays(tse_topic)$counts %>% t() %>% as.data.frame() -> otu
```

```
lda_k2 <- LDA((otu), k = 2, method = "VEM", control = list(seed = 1406L))

library(tidytext)
b_df <- data.frame(tidy(lda_k2, matrix = "beta")) %>%
  pivot_wider(names_from = "topic",values_from = "beta",names_prefix = "Topic")
g_df <- data.frame(tidy(lda_k2, matrix = "gamma")) %>%
  arrange(document, topic) %>%
  pivot_wider(names_from = "topic",values_from = "gamma",names_prefix = "Topic")
```

Load validation data

Healthy topics

```
# Jun-Pei healthy
junpei <- readRDS("Data/JunPei_28_healthy.RDS") %>%
  mia::makeTreeSummarizedExperimentFromPhyloseq()

# Junpei Perio
junpei_perio <- readRDS("Data/JunPei_Perio.RDS") %>%
  mia::makeTreeSummarizedExperimentFromPhyloseq()

# Griffen A
griffen_a <- readRDS("Data/Griffen_a_healthy.RDS") %>%
  mia::makeTreeSummarizedExperimentFromPhyloseq()

# Griffen B
griffen_b <- readRDS("Data/Griffen_b_healthy.RDS") %>%
  mia::makeTreeSummarizedExperimentFromPhyloseq()

# Orami
orami <- readRDS("Data/orami_healthy.RDS") %>%
  mia::makeTreeSummarizedExperimentFromPhyloseq()
```

Veryify fitted model on external data

```
refit_Model <- function(orig_model,new_data_tse) {

  ## Prepare genus_species names
  rowData(new_data_tse)[,7] <- paste0(rowData(new_data_tse)[,6],"_",rowData(new_data_tse)[,7])

  ## Agglomerate on Species Rank
  topic_tse <- mia::agglomerateByRank(new_data_tse,"Species",onRankOnly=T)

  ## Extract assay
  assays(topic_tse)$counts %>% t() %>% as.data.frame() -> otu

  ## Fit previously estimated model to new data
  fit <- LDA(otu,
                    k = 2,
```

```r
                      method = "VEM",
                      model = orig_model, # Define previous model
                      control = list(seed = 1406L,
                                     estimate.beta = FALSE) # Betas are used from old model
              )

  ## Prepare DF with beta coefficients - should be equal to original model
  b_df_fit <- data.frame(tidy(fit, matrix = "beta")) %>%
    mutate(topic = ifelse(topic == 1, "Pathogenic","Healthy")) %>%
    dplyr::rename("Taxon" = "term") %>%
    pivot_wider(names_from = "topic",
                values_from = "beta")

  g_df_fit <- data.frame(tidy(fit, matrix = "gamma")) %>%
    arrange(document, topic) %>%
    mutate(topic = ifelse(topic == 1, "Pathogenic","Healthy")) %>%
    pivot_wider(names_from = "topic",
                values_from = "gamma") %>%
    dplyr::rename("Sample" = "document")


  ## Prepare output
  out <- (list("Model" = fit,
               "betas" = b_df_fit,
               "gammas" = g_df_fit))

  return(out)

}
```

```r
orami_fit <- refit_Model(lda_k2,orami)
griffen_a_fit <- refit_Model(lda_k2,griffen_a)
griffen_b_fit <- refit_Model(lda_k2,griffen_b)
junpei_fit <- refit_Model(lda_k2,junpei)
junpei_perio_fit <- refit_Model(lda_k2,junpei_perio)

# Original data at baseline
abparo_fit_v2_placebo <-
  refit_Model(lda_k2,tse[,colData(tse)$visit == "V2" & colData(tse)$Treatment_Group == "placebo"])
abparo_fit_v2_anti <-
  refit_Model(lda_k2,tse[,colData(tse)$visit == "V2" & colData(tse)$Treatment_Group == "antibiotic"])
abparo_fit <- refit_Model(lda_k2,tse)
```

```r
plotTopicDistribution <- function(df) {
  p <- df$gammas %>%
  pivot_longer(cols = c("Pathogenic","Healthy"),
               names_to = "Topic",
               values_to = "Loading") %>%
  ggplot(aes(x = Sample, y = Loading, fill = Topic)) +
  geom_bar(stat="identity",position = "fill")  +
  ggpubr::theme_pubclean() +
  theme(axis.text.x = element_blank(),
        axis.ticks.x = element_blank(),
```

```
        legend.position = "left") +
    ylab("Topic loading") +
    xlab(NULL) +
    scale_fill_manual(labels = c("Normobiotic","Dysbiotic"),
                      values = c("#00CD00","#EE0000"))

    return(p)
}


plotTopicContribution <- function(df,thresh = 0.01) {

   p <-  df$betas  %>%
  pivot_longer(cols = c("Pathogenic","Healthy"),
               names_to = "Topic",
               values_to = "Contribution") %>%
  filter(Contribution > thresh) %>%
  mutate(Contribution = ifelse(Topic == "Pathogenic", Contribution*(-1),Contribution)) %>%
    ggplot(aes(x = Taxon, y = Contribution)) +
    geom_segment(aes(yend = 0,xend=reorder(Taxon,-Contribution)),size=1,color = "grey") +
    geom_hline(yintercept = 0) +
    geom_point(size = 1,aes(color = Topic)) +
    ggpubr::theme_pubclean() +
    theme(axis.text.y = element_text(size = 5)) +
    coord_flip() +
    theme(legend.position = "bottom") +
    ylab("Relative topic contribution") +
    xlab("Species") +
    scale_color_manual(labels = c("Normobiotic","Dysbiotic"),
                       values = c("#00CD00","#EE0000"))
  return(p)
}
```

```
plotTopicDistribution(abparo_fit_v2_placebo) + ggtitle("ABParo baseline placebo") +
plotTopicDistribution(abparo_fit_v2_anti) + ggtitle("ABParo baseline antibiotics") +
plotTopicDistribution(orami_fit) + ggtitle("Internal validation") +
  plotTopicDistribution(junpei_fit) + ggtitle("Pei et al. 2020") +
  plotTopicDistribution(griffen_a_fit) + ggtitle("Griffen et al. 2021 (a)") +
  plotTopicDistribution(griffen_b_fit) + ggtitle("Griffen et al. 2021 (b)") +
  plot_layout(guides = "collect",ncol = 2) + plot_annotation(title = "Model validation") -> p2
```

```
p1 <- plotTopicContribution(orami_fit) +
  theme(axis.text.y = element_text(size=10),
        legend.position = "none")
```

```
p1 + p2 + plot_layout(guides = "collect",widths = c(2,6))
```

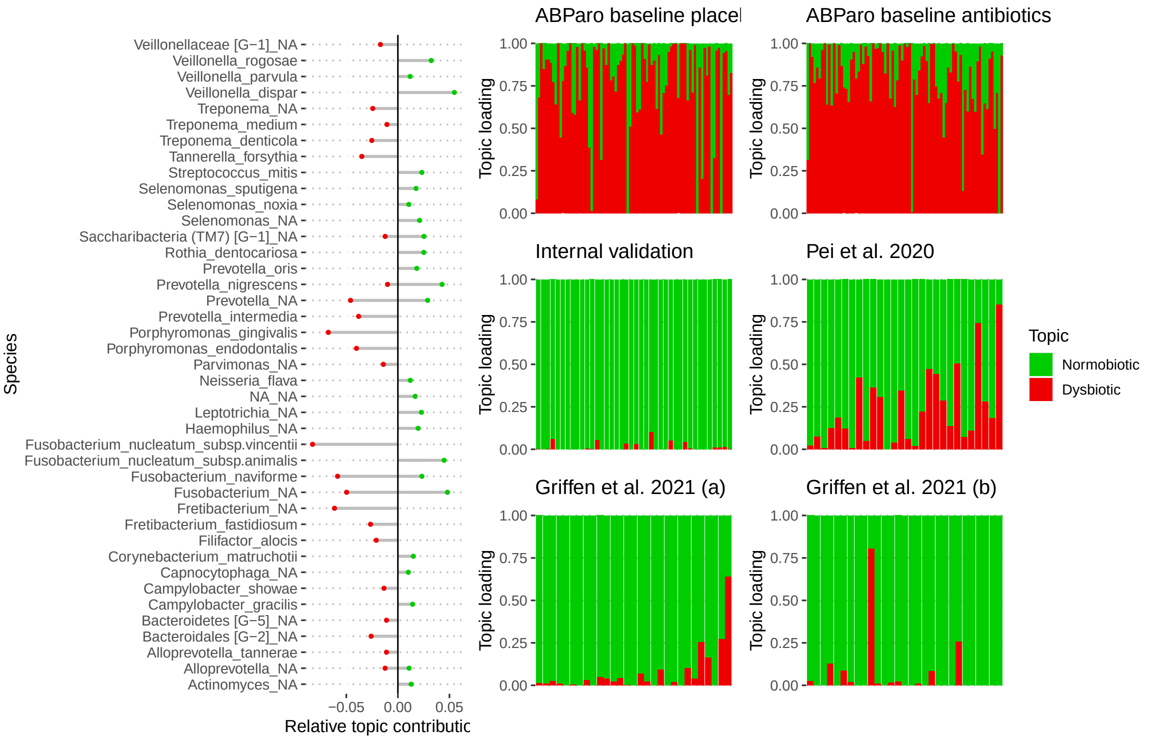


```r
p <- p1 + p2 + plot_layout(guides = "collect",widths = c(2,6))

ggsave("Plots/topic_summary.tiff",plot = p,device = "tiff",width = 12,height = 8, scale = 1.2)
```

Treatment effect on dysbiosis

```r
colData(tse) %>%
  as.data.frame() %>%
  dplyr::select(visit,Treatment_Group) %>%
  rownames_to_column("Sample") %>%
  dplyr::left_join(abparo_fit$gammas) %>%
  group_by(visit,Treatment_Group) %>%
  dplyr::summarize(Pathogenic = median(Pathogenic),
            Healthy = median(Healthy)) %>%
    mutate(across(where(is.numeric),function(x) round(x,4)*100)) %>%
  kbl() %>%
 kable_styling(latex_options = c("striped","HOLD_position"))
```

visit	Treatment_Group	Pathogenic	Healthy
V2	placebo	87.45	12.55
V2	antibiotic	86.02	13.98
V4	placebo	61.21	38.79
V4	antibiotic	2.52	97.48
V6	placebo	71.09	28.91
V6	antibiotic	34.57	65.43
V8	placebo	80.72	19.28
V8	antibiotic	52.53	47.47
V12	placebo	82.54	17.46
V12	antibiotic	48.86	51.14

```
topic_rel <- abparo_fit$gammas
colnames(topic_rel)[2:3] <- paste0(colnames(topic_rel)[2:3] ,"_rel")

colData(tse) <- colData(tse) %>%
  as.data.frame() %>%
  rownames_to_column("Sample") %>%
  dplyr::left_join(topic_rel) %>%
  DataFrame()
```

Association with clinical outcomes

```
dichotomizeMedian <- function(x) {ifelse(x>median(x,na.rm=T),1,0)}

colData(tse) %>%
  as.data.frame() %>%
  dplyr::select(visit,subject,Treatment_Group,SEX,SMOKING,AGE,Number_Teeth,
                Anteil_Attachment_Loss,Anteil_Pocket_Depth,Bleeding_on_Probing,
                Pathogenic_rel, Healthy_rel,SMDI,CO.measurements.ppm.,seq.depth) %>%
   mutate(time = case_when(visit == "V2"~0,
                           visit == "V4"~2,
                           visit == "V6"~8,
                           visit == "V8"~14,
                           visit == "V12"~26)) %>%
  mutate(Pathogenic = round(Pathogenic_rel*seq.depth,0),
         Healthy = round(Healthy_rel*seq.depth,0)) %>%
  rownames_to_column("Sample") %>%
  #dplyr::left_join(topic_rel) %>%
  mutate(topic_bal = log(Pathogenic_rel / Healthy_rel),
         dominant_topic = ifelse(Pathogenic > Healthy, "Pathogenic","Healthy")) -> model_df
```

```
p <- model_df %>%
  ggplot(aes(x = time,fill = Treatment_Group,color = Treatment_Group, y = topic_bal)) +
  geom_smooth() +
  scale_fill_d3() +
  scale_color_d3() +
    labs(y = "Topic balance",
       x = "Months",fill = "Treatment Group", color = "Treatment Group") +
  theme_pubr() +
  theme(legend.position = "bottom")
```

```
p
```

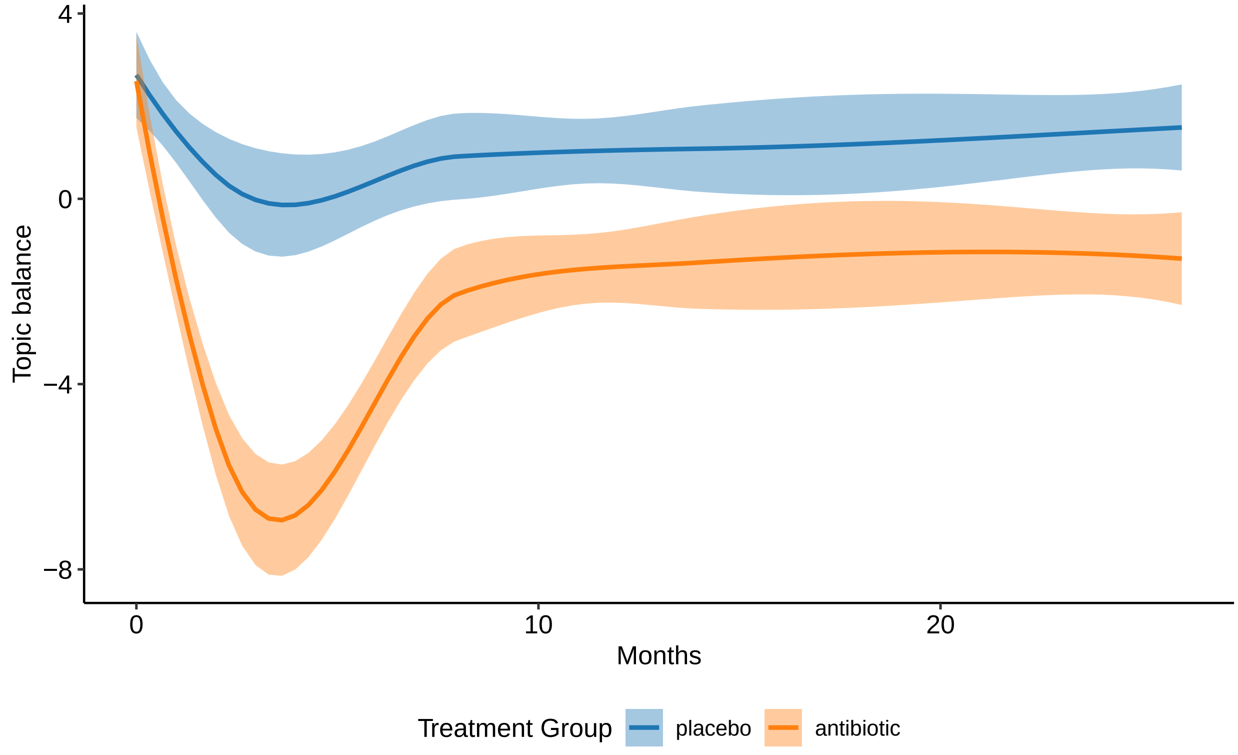


```
ggsave("Plots/dysbiosis_longitudinal.tiff",plot = p,device = "tiff",width = 8,height = 5,dpi = 300,unit
```

```
model_df %>%
  group_by(visit) %>%
  mutate(AL.median = dichotomizeMedian(Anteil_Attachment_Loss),
         PD.median = dichotomizeMedian(Anteil_Pocket_Depth),
         BP.median = dichotomizeMedian(Bleeding_on_Probing),
         BP.ttt = case_when(Bleeding_on_Probing <= 10 ~ 0,
                            Bleeding_on_Probing > 10 & Bleeding_on_Probing <= 20 ~ 1,
                            Bleeding_on_Probing > 20 ~ 2),
         PD.abs= round(((Number_Teeth*6)/100) *  Anteil_Pocket_Depth,0),
         PD.ttt = ifelse(PD.abs <= 4,0,1)) %>%
  ungroup() -> model_df
```

```
fit_models <- function(data = model_df,
                       curr_visit = "V12") {

  # Select outcome timepoint
  curr_df <- data %>% filter(visit == curr_visit)

  if(curr_visit != "V2") {
  fit_AL1 <- glm(AL.median ~ topic_bal,data=curr_df,family = "binomial")
  fit_AL2 <- glm(AL.median ~ topic_bal+SEX+SMOKING+AGE+Treatment_Group,
                 data=curr_df,family = "binomial")
```

```
  } else {
    fit_AL1 <- "Skipped"
    fit_AL2 <- "Skipped"
  }
  fit_PD1 <- glm(PD.median ~ topic_bal,data=curr_df,family = "binomial")
  fit_PD2 <- glm(PD.median ~ topic_bal+SEX+SMOKING+AGE+Treatment_Group,
                 data=curr_df,family = "binomial")

  fit_BP1 <- glm(BP.median ~ topic_bal,data=curr_df,family = "binomial")
  fit_BP2 <- glm(BP.median ~ topic_bal+SEX+SMOKING+AGE+Treatment_Group,
                 data=curr_df,family = "binomial")

  out <- list(fit_AL1,
              fit_AL2,
              fit_PD1,
              fit_PD2,
              fit_BP1,
              fit_BP2)

}


models_v2 <- fit_models(data = model_df, curr_visit = "V2")
models_v4 <- fit_models(data = model_df, curr_visit = "V4")
models_v6 <- fit_models(data = model_df, curr_visit = "V6")
models_v8 <- fit_models(data = model_df, curr_visit = "V8")
models_v12 <- fit_models(data = model_df, curr_visit = "V12")
```

```
sjPlot::tab_model(models_v2[[4]],
               models_v4[[4]],
               models_v12[[4]],title = "PPD 5mm") %>%
  sjtable2df::mtab2df(n_models = 3) %>%
  kable(booktabs = T,caption = "Percentage PPD 5mm") %>%
   kable_styling(latex_options = c("striped","HOLD_position","scale_down")) %>%
   add_header_above(c(" " = 1, "pre-treatment" = 3, "2 months" = 3, "26 months" = 3))
```

Table 1: Percentage PPD 5mm

	pre-treatment			2 months			26 months		
Predictors	Odds Ratios	CI	p	Odds Ratios	CI	p	Odds Ratios	CI	p
(Intercept)	0.30	0.10 – 0.84	0.026	1.56	0.59 – 4.20	0.368	0.78	0.30 – 2.03	0.617
topic bal	1.26	1.13 – 1.43	<0.001	1.12	1.03 – 1.23	0.007	1.14	1.04 – 1.27	0.011
SEX [male]	2.92	1.48 – 5.94	0.002	2.82	1.42 – 5.76	0.004	2.02	1.03 – 4.05	0.042
SMOKING [Smoker]	1.16	0.55 – 2.48	0.695	0.82	0.37 – 1.81	0.629	1.97	0.93 – 4.27	0.079
AGE [45<55]	1.09	0.43 – 2.77	0.860	0.54	0.21 – 1.34	0.189	0.66	0.26 – 1.63	0.373
AGE [>55]	0.79	0.29 – 2.19	0.650	0.43	0.16 – 1.16	0.100	0.81	0.31 – 2.11	0.658
Treatment Group[antibiotic]	1.08	0.55 – 2.14	0.826	0.53	0.24 – 1.17	0.117	0.51	0.25 – 1.02	0.058
Observations	163			163			163		
R2 Tjur	0.176			0.180			0.156		

```
sjPlot::tab_model(models_v2[[6]],
               models_v4[[6]],
               models_v12[[6]],title = "Bleeding") %>%
```

```
  sjtable2df::mtab2df(n_models = 3) %>%
  kable(booktabs = T,caption = "Percentage Bleeding") %>%
   kable_styling(latex_options = c("striped","HOLD_position","scale_down")) %>%
   add_header_above(c(" " = 1, "pre-treatment" = 3, "2 months" = 3, "26 months" = 3))
```

Table 2: Percentage Bleeding

	pre-treatment			2 months			26 months		
Predictors	Odds Ratios	CI	p	Odds Ratios	CI	p	Odds Ratios	CI	p
(Intercept)	1.35	0.53 – 3.49	0.528	3.02	1.14 – 8.46	0.029	1.98	0.79 – 5.22	0.153
topic bal	1.08	1.00 – 1.18	0.053	1.09	1.01 – 1.19	0.034	1.08	0.99 – 1.18	0.095
SEX [male]	0.91	0.48 – 1.71	0.773	0.76	0.39 – 1.47	0.410	1.20	0.63 – 2.31	0.576
SMOKING [Smoker]	0.53	0.25 – 1.08	0.084	0.66	0.30 – 1.44	0.301	0.91	0.43 – 1.90	0.801
AGE [45<55]	0.78	0.33 – 1.81	0.557	0.87	0.35 – 2.11	0.756	0.49	0.20 – 1.17	0.113
AGE [>55]	0.56	0.22 – 1.39	0.214	0.54	0.20 – 1.44	0.224	0.65	0.25 – 1.65	0.365
Treatment Group[antibiotic]	1.09	0.58 – 2.05	0.795	0.42	0.19 – 0.90	0.026	0.45	0.23 – 0.88	0.021
Observations	163			163			163		
R2 Tjur	0.038			0.146			0.089		

```
sjPlot::tab_model(models_v4[[2]],
                models_v12[[2]],title = "%CAL1.3mm") %>%
  sjtable2df::mtab2df(n_models = 2) %>%
  kable(booktabs = T,caption = "Percentage CAL1.3mm") %>%
   kable_styling(latex_options = c("striped","HOLD_position","scale_down")) %>%
   add_header_above(c(" " = 1,"2 months" = 3, "26 months" = 3))
```

Table 3: Percentage CAL1.3mm

	2 months			26 months		
Predictors	Odds Ratios	CI	p	Odds Ratios	CI	p
(Intercept)	0.33	0.12 – 0.86	0.028	0.55	0.21 – 1.40	0.212
topic bal	1.05	0.97 – 1.14	0.277	1.11	1.02 – 1.23	0.027
SEX [male]	0.73	0.37 – 1.41	0.351	1.00	0.52 – 1.93	0.998
SMOKING [Smoker]	2.86	1.32 – 6.39	0.008	2.63	1.25 – 5.69	0.012
AGE [45<55]	3.24	1.32 – 8.44	0.013	0.90	0.37 – 2.19	0.817
AGE [>55]	2.27	0.85 – 6.37	0.110	1.77	0.69 – 4.69	0.237
Treatment Group[antibiotic]	0.72	0.32 – 1.62	0.432	0.82	0.41 – 1.64	0.581
Observations	163			163		
R2 Tjur	0.123			0.109		

```
fit <- glm(PD.ttt ~ topic_bal+Treatment_Group+SEX+SMOKING+AGE,data = model_df,
          family = "binomial")
```

```
sjPlot::tab_model(fit) %>%
  sjtable2df::mtab2df(n_models = 1) %>%
  kable(booktabs = T,
        caption = "Treat-to-target endpoint as <= 4 sites with PPD >= 5mm 26 months after treatment") %
   kable_styling(latex_options = c("striped","HOLD_position","scale_down"))
```

Table 4: Treat-to-target endpoint as <= 4 sites with PPD >= 5mm 26 months after treatment

Predictors	Odds Ratios	CI	p
(Intercept)	4.05	2.52 – 6.62	<0.001
topic bal	1.10	1.06 – 1.14	<0.001
Treatment Group[antibiotic]	0.42	0.30 – 0.58	<0.001
SEX [male]	1.31	0.95 – 1.80	0.100
SMOKING [Smoker]	1.28	0.89 – 1.83	0.186
AGE [45<55]	0.66	0.42 – 1.02	0.066
AGE [>55]	0.72	0.44 – 1.14	0.167
Observations	812		
R2 Tjur	0.115		

Clustering of responder categories

```
model_df %>%
  dplyr::select(subject,visit,dominant_topic) %>%
  pivot_wider(names_from = "visit", values_from = "dominant_topic") %>%
  column_to_rownames("subject") %>%
  arrange(V2,V4,V6,V8,V12) %>%
  mutate(across(everything(),\(x) ifelse(x == "Healthy",1,2))) %>%
  as.matrix() -> mat

# calculate distance
d_dist <- cluster::daisy(mat, metric = "gower", weights =c(1))

# hierarchical clustering
hc <- hclust(d_dist, method = "complete")
cluster <- cutree(hc, k=20)

cluster[cluster %in% c(9,7,11)] <- 13 #short term responder
cluster[cluster %in% c(3,4,5)] <- 2 # indifferent pre normobitoic
cluster[cluster %in% c(10,12,14,15,16,17,18,19)] <- 8 # indifferent pre dysbiotic

cluster[cluster == 13] <- "short-term responder"
cluster[cluster == 1] <- "long-term responder"
cluster[cluster == 6] <- "long-term responder"
cluster[cluster == 20] <- "non-responder"
cluster[cluster == 2] <-  "indifferent"
cluster[cluster == 8] <-  "indifferent"

cluster_df <- as.data.frame(cluster) %>%
  rownames_to_column("subject")
```

```r
mat <- mat[names(cluster),]
mat <- ifelse(mat==1,"Normobiotic","Dysbiotic")
```

```r
col_fun <- circlize::colorRamp2(c(1,.5, 0), c("#00CD00","orange", "#EE0000"))

ht_df <- model_df %>%
  dplyr::select(subject,visit,Healthy_rel,Treatment_Group,
              Anteil_Attachment_Loss,Anteil_Pocket_Depth,
              Bleeding_on_Probing) %>%
  dplyr::left_join(cluster_df, by = "subject") %>%
  mutate(cluster = case_when(cluster == "non-responder" ~ "0: non-responder",
                             cluster == "indifferent" ~ "1: indifferent",
                             cluster == "short-term responder" ~ "2: short-term responder",
                             cluster == "long-term responder" ~ "3: responder",
                             .ptype = "factor"),
         visit = case_when(visit == "V2" ~ "pre-treatment",
                           visit == "V4" ~ "2 months",
                           visit == "V6" ~ "8 months",
                           visit == "V8" ~ "14 months",
                           visit == "V12" ~ "26 months",.ptype = "factor")) %>%
  mutate(cluster = factor(cluster, levels = c("0: non-responder",
                          "1: indifferent",
                          "2: short-term responder",
                          "3: responder")),
         visit = factor(visit, levels = c("pre-treatment",
                                          "2 months",
                                          "8 months",
                                          "14 months",
                                          "26 months")))



param_df <- ht_df %>%
  pivot_longer(cols = c(Anteil_Attachment_Loss,
                        Anteil_Pocket_Depth,
                        Bleeding_on_Probing),
               names_to = "Parameter") %>%
  group_by(visit, cluster, Treatment_Group, Parameter) %>%
  summarise(avg = mean(value,na.rm = T),
            sd = sd(value,na.rm=T),
            N = n()) %>%
  ungroup() %>%
  mutate(t.score = qt(0.05/2,df = N-1,lower.tail = F),
         se = sd/(N-1),
         margin.error = t.score * se,
         lower = avg - margin.error,
         upper = avg + margin.error) %>%
  mutate(Parameter = case_when(
    Parameter == "Anteil_Attachment_Loss" ~ "%CAL1.3mm",
    Parameter == "Anteil_Pocket_Depth" ~ "%PPD5mm",
    Parameter == "Bleeding_on_Probing" ~ "%Bleeding"
  ))
```

```r
param_plot_df <- model_df %>%
  dplyr::left_join(data.frame("cluster" = cluster) %>%
                     rownames_to_column("subject")) %>%
  dplyr::select(time,cluster,Treatment_Group,Anteil_Attachment_Loss,
                Anteil_Pocket_Depth,Bleeding_on_Probing) %>%
  mutate(cluster = make.names(cluster),
         cluster = ordered(cluster,levels = c("non.responder",
                                              "indifferent",
                                              "short.term.responder",
                                              "long.term.responder"))) %>%
  pivot_longer(cols = c(Anteil_Attachment_Loss,
                        Anteil_Pocket_Depth,
                        Bleeding_on_Probing),
               names_to = "Parameter") %>%
  ungroup() %>%
   group_by(time, cluster, Treatment_Group, Parameter) %>%
  summarise(avg = mean(value,na.rm = T),
            sd = sd(value,na.rm=T),
            N = n()) %>%
  ungroup() %>%
  mutate(t.score = qt(0.05/2,df = N-1,lower.tail = F),
         se = sd/(N-1),
         margin.error = t.score * se,
         lower = avg - margin.error,
         upper = avg + margin.error) %>%
  mutate(Parameter = case_when(
    Parameter == "Anteil_Attachment_Loss" ~ "%CAL1.3mm",
    Parameter == "Anteil_Pocket_Depth" ~ "%PPD5mm",
    Parameter == "Bleeding_on_Probing" ~ "%Bleeding"
  )) %>%
  mutate(time = ifelse(time == 0, -2, time))


gg_ht <- ht_df %>%
  ggplot(aes(x = visit, y = subject, fill = Healthy_rel)) +
  geom_tile(color = "black") +
  facet_wrap(~cluster, scales = "free_y", ncol = 1, strip.position = "left") +
  ggdist::theme_tidybayes() +
  scale_fill_gradient2(midpoint = .5,
                       high = scales::muted("#00CD00"),
                       mid = "orange",
                       low = scales::muted("#EE0000")) +
  theme(
    strip.background = element_blank(), # Removes background of facet labels
    #strip.placement = "outside", # Places the labels outside of the plot area
    panel.spacing = unit(1, "mm"), # Reduces spacing between facets
    axis.text.y = element_blank(), # Hides y-axis text
    axis.ticks.y = element_blank(), # Hides y-axis ticks
    plot.margin = unit( c(0,0,0,0) , units = "lines"),
    strip.text.y = element_blank() ,
    legend.position = "bottom",
    axis.title.y = element_blank(),
    axis.title.x = element_blank()
```

```
  ) +
  labs(fill = "Normobiosis")

gg_ht_treat <- ht_df %>%
  filter(visit == "pre-treatment") %>%
  ggplot(aes(x = visit, y = subject, fill = Treatment_Group)) +
  facet_wrap(~cluster, scales = "free_y", ncol = 1, strip.position = "left") +
  geom_tile(color = "white") +
  scale_fill_manual(values = c("black","grey")) +
  theme_void() +
  theme(strip.text.y = element_text(angle = 90,size = 8) ,
  #strip.background = element_blank(),
  plot.margin = unit( c(0,0,0,0) , units = "lines" ),
  legend.position = "bottom") +
  labs(fill = "Treatment group")


p_cal <- param_plot_df %>%
  filter(Parameter == "%CAL1.3mm") %>%
  #filter(cluster == "non.responder") %>%
  ggplot(aes(x=time, y = avg,fill=Treatment_Group,color = Treatment_Group)) +
   geom_line(aes(color = Treatment_Group,group = Treatment_Group)) +
  geom_point() +
  geom_errorbar(aes(ymin = lower, ymax = upper)) +
  facet_wrap(~cluster,ncol = 1) +
  scale_color_d3() +
  theme(legend.position = "bottom") +
  #ggdist::theme_ggdist() +
  theme_classic() +
  theme(legend.position = "none",
        axis.title.x = element_blank(),
        axis.title.y = element_blank(),
        axis.text.x = element_text(angle = 45, vjust = 1,hjust = 1),
        strip.text.x = element_blank(),
        strip.text.y = element_blank(),
        strip.background = element_blank(),
  plot.margin = unit( c(0,0,0,0) , units = "cm" )) +
  scale_x_continuous(breaks = c(-2,2,8,14,26),labels = c("pre","2 months",
                                                         "8 months",
                                                         "14 months",
                                                         "26 months")) +
    scale_y_continuous(expand = c(.1,0)) +
  labs(caption = "%CAL1.3mm")

p_ppd <- param_plot_df %>%
  filter(Parameter == "%PPD5mm") %>%
  #filter(cluster == "non.responder") %>%
  ggplot(aes(x=time, y = avg,fill=Treatment_Group,color = Treatment_Group)) +
   geom_line(aes(color = Treatment_Group,group = Treatment_Group)) +
  geom_point() +
  geom_errorbar(aes(ymin = lower, ymax = upper)) +
  facet_wrap(~cluster,ncol = 1) +
  scale_color_d3() +
```

```r
  theme(legend.position = "bottom") +
  #ggdist::theme_ggdist() +
  theme_classic() +
  theme(legend.position = "none",
        axis.title.x = element_blank(),
        axis.title.y = element_blank(),
        axis.text.x = element_text(angle = 45, vjust = 1,hjust = 1),
        strip.text.x = element_blank(),
        strip.text.y = element_blank(),
        strip.background = element_blank(),
  plot.margin = unit( c(0,0,0,0) , units = "cm" )) +
  scale_x_continuous(breaks = c(-2,2,8,14,26),labels = c("pre","2 months",
                                                       "8 months",
                                                       "14 months",
                                                       "26 months")) +
    scale_y_continuous(expand = c(.2,0)) +
  labs(caption = "%PPD5mm")

p_bleed <- param_plot_df %>%
  filter(Parameter == "%Bleeding") %>%
  ggplot(aes(x=time, y = avg,fill=Treatment_Group,color = Treatment_Group)) +
   geom_line(aes(color = Treatment_Group,group = Treatment_Group)) +
  geom_point() +
  geom_errorbar(aes(ymin = lower, ymax = upper)) +
  facet_wrap(~cluster,ncol = 1) +
  scale_color_d3() +
  theme(legend.position = "bottom") +
  theme_classic() +
  theme(legend.position = "none",
        axis.title.x = element_blank(),
        axis.title.y = element_blank(),
        axis.text.x = element_text(angle = 45, vjust = 1,hjust = 1),
        strip.text.x = element_blank(),
        strip.text.y = element_blank(),
        strip.background = element_blank(),
  plot.margin = unit( c(0,0,0,0) , units = "cm" )) +
  scale_x_continuous(breaks = c(-2,2,8,14,26),labels = c("pre","2 months",
                                                       "8 months",
                                                       "14 months",
                                                       "26 months")) +
  scale_y_continuous(expand = c(.2,0)) +
  labs(caption = "%Bleeding")



p <- gg_ht_treat + plot_spacer() + gg_ht + p_cal + p_ppd + p_bleed +
  plot_layout(widths = c(.5,-1.6,10,5,5,5),
            guides = "collect") & theme(legend.position = "top")

ggsave(plot = p, filename = "Plots/ggplot_heatmap.tiff",
       width = 12,
       height = 8,
       device = "tiff",
```

```
       dpi = 300)
```

```
p
```

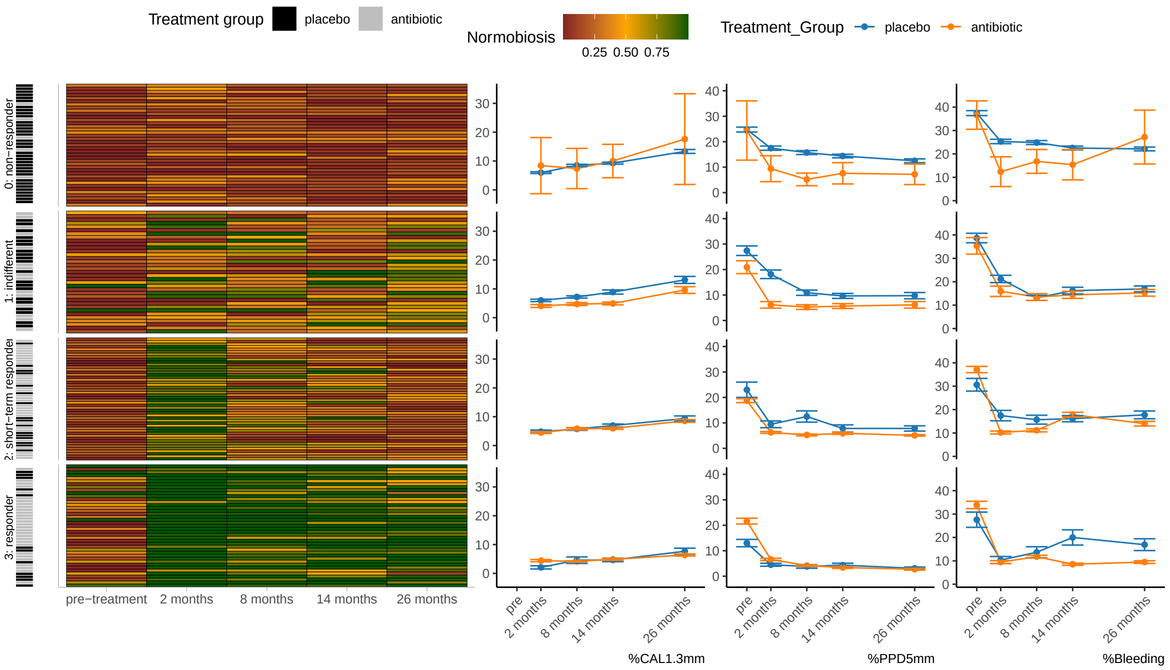


```
p_cal <- param_plot_df %>%
  filter(Parameter == "%CAL1.3mm") %>%
  #filter(cluster == "non.responder") %>%
  ggplot(aes(x=time, y = avg,fill=Treatment_Group,color = Treatment_Group)) +
   geom_line(aes(color = Treatment_Group,group = Treatment_Group)) +
  geom_point() +
  geom_errorbar(aes(ymin = lower, ymax = upper)) +
  facet_wrap(~cluster,ncol = 1, strip.position = "left") +
  scale_color_d3() +
  theme(legend.position = "bottom") +
  #ggdist::theme_ggdist() +
  theme_classic() +
  theme(legend.position = "none",
        axis.title.x = element_blank(),
        axis.title.y = element_blank(),
        strip.placement = "left",
  plot.margin = unit( c(0,0,0,0) , units = "cm" )) +
  labs(caption = "%CAL1.3mm")

p_ppd <- param_plot_df %>%
  filter(Parameter == "%PPD5mm") %>%
  #filter(cluster == "non.responder") %>%
```

```r
  ggplot(aes(x=time, y = avg,fill=Treatment_Group,color = Treatment_Group)) +
   geom_line(aes(color = Treatment_Group,group = Treatment_Group)) +
  geom_point() +
  geom_errorbar(aes(ymin = lower, ymax = upper)) +
  facet_wrap(~cluster,ncol = 1, strip.position = "left") +
  scale_color_d3() +
  theme(legend.position = "bottom") +
  #ggdist::theme_ggdist() +
  theme_classic() +
  theme(legend.position = "none",
        axis.title.x = element_blank(),
        axis.title.y = element_blank(),
        strip.text.x = element_blank(),
        strip.text.y = element_blank(),
        strip.background = element_blank(),
                strip.placement = "left",
  plot.margin = unit( c(0,0,0,0) , units = "cm" )) +
  labs(caption = "%PPD5mm")

p_bleed <- param_plot_df %>%
  filter(Parameter == "%Bleeding") %>%
  #filter(cluster == "non.responder") %>%
  ggplot(aes(x=time, y = avg,fill=Treatment_Group,color = Treatment_Group)) +
   geom_line(aes(color = Treatment_Group,group = Treatment_Group)) +
  geom_point() +
  geom_errorbar(aes(ymin = lower, ymax = upper)) +
  facet_wrap(~cluster,ncol = 1, strip.position = "left") +
  scale_color_d3() +
  theme(legend.position = "bottom") +
  #ggdist::theme_ggdist() +
  theme_classic() +
  theme(legend.position = "none",
        axis.title.x = element_blank(),
        axis.title.y = element_blank(),
        strip.text.x = element_blank(),
        strip.text.y = element_blank(),
        strip.background = element_blank(),
                strip.placement = "left",
  plot.margin = unit( c(0,0,0,0) , units = "cm" )) +
  labs(caption = "%Bleeding")
```

```r
p2 <- p_cal + p_ppd + p_bleed + plot_layout(guides = "collect") & theme(legend.position = "top")
ggsave(filename = "Plots/treatment_outcome.PDF", p2,width = 10,height = 8, device = "pdf")
```

Analysis of main outcome

DAG representation of causal intervention effect

```r
library(ggdag)
library(dagitty)
```

```r
df <- data.frame(name = c("AGE","AL","RP","Rand","SEX","SMOKING","TG")) %>%
  mutate(x = case_when(name == "AGE" ~ 3,
                       name == "SEX" ~ 4,
                       name == "SMOKING" ~ 5,
                       name == "AL" ~ 5,
                       name == "RP" ~ 2.5,
                       name == "Rand" ~ -2,
                       name == "TG" ~ 0),
         y = case_when(name %in% c("AGE","SEX","SMOKING") ~ 3,
                       name == "AL" ~ 0,
                       name == "RP" ~ 1,
                       name == "Rand" ~ 0,
                       name == "TG" ~ 0))

dag <- dagify(AL ~ TG + RP,
       RP ~ TG,
       TG ~ Rand,
       AL ~ SEX + AGE + SMOKING,
       RP ~ SEX + AGE + SMOKING,
       labels = c("SEX" = "Sex",
                  "SMOKING" = "Smoking",
                  "TG" = "Treatment",
                  "AGE" = "Age",
                  "Rand" = "Randomization",
                  "AL" = "Attachment Loss",
                  "RP" = "Response pattern"),
       exposure = "TG",
       outcome = "AL",
       coords = df
)
```

```r
ggdag_status(dag, stylized = F,text_col = "white")  +
  theme_dag()
```

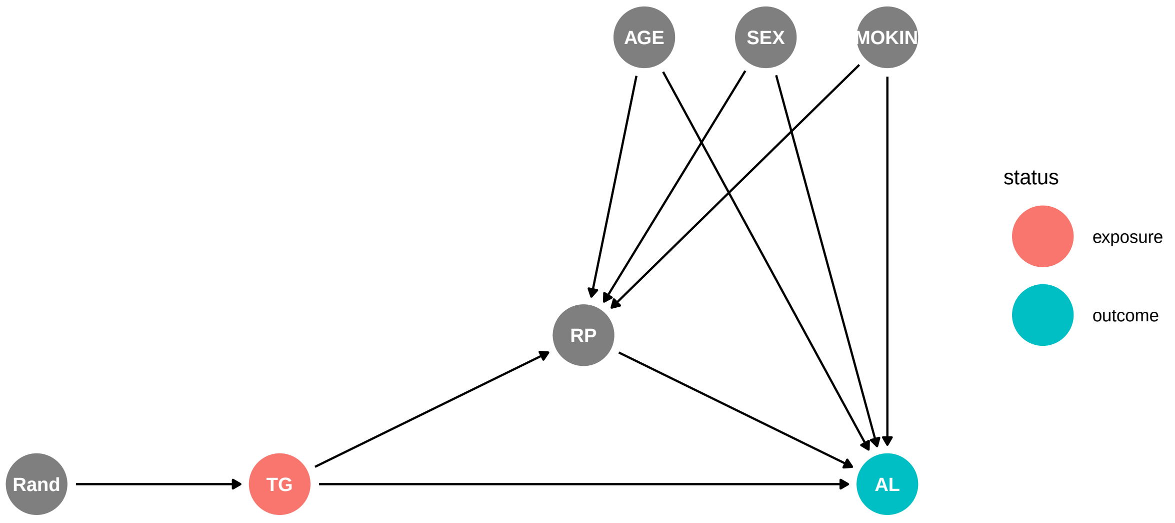

```r
adjustmentSets(dag)
```

```
##  {}
```

Mediation analysis

Total effect of antibiotic treatment

```r
model_df %>%
  dplyr::left_join(cluster_df, by = "subject") %>%
  mutate(Number_Sites = Number_Teeth*6,
    Absolute_Attachment_Loss = round((Number_Sites/100)*Anteil_Attachment_Loss,0)) %>%
    mutate(pattern = make.names(cluster),
           pattern_ordered = ordered(pattern,levels = c("non.responder",
                                                        "indifferent",
                                                        "short.term.responder",
                                                        "long.term.responder"))) -> model_df
```

```r
model_df %>%
ggplot(aes(x = pattern_ordered, y = Absolute_Attachment_Loss, fill = pattern_ordered)) +
  geom_boxplot() +
  theme_minimal() +
  theme(axis.text.x = element_text(angle = 45,vjust = .7,hjust =.8)) +
  scale_y_log10()
```

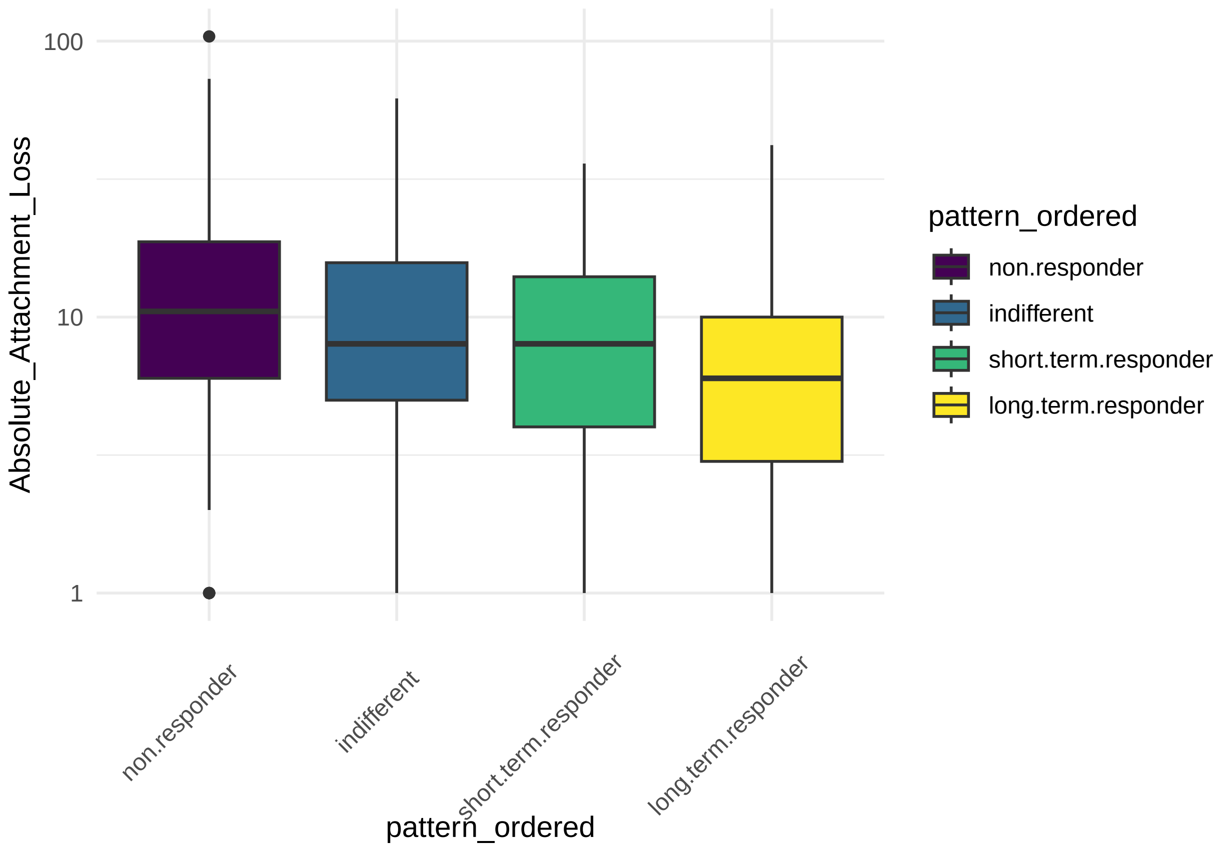


```
fit_total <- brm(
  Absolute_Attachment_Loss ~ 0 + Intercept + Treatment_Group + offset(log(Number_Sites)),
  data = model_df %>% filter(visit == "V12"),
  family = negbinomial,cores = 4,chains = 4)
```

Direct effect

The direct effect is the effect of the intervention that is on top of the effect that goes through measured oral microbiome changes.

```
fit_direct <- brm(Absolute_Attachment_Loss ~ Treatment_Group + pattern_ordered + AGE + SEX + SMOKING +
                    data = model_df %>% filter(visit == "V12"),
                    family = negbinomial,cores = 4,chains = 4)
```

```
total <- brms::posterior_samples(fit_total,pars = "Treatment_Groupantibiotic")
direct <- brms::posterior_samples(fit_direct,pars = "Treatment_Groupantibiotic")

indirect <- total - direct

mean_effects_df <- bind_rows(mean_qi(exp(total)),
          mean_qi(exp(direct)),
          mean_qi(exp(indirect))) %>%
  mutate(Effect = c("Total","Direct","Indirect"), .before = 1)
```

```r
median_effects_df <- bind_rows(median_qi(exp(total)),
          median_qi(exp(direct)),
          median_qi(exp(indirect))) %>%
  mutate(Effect = c("Total","Direct","Indirect"), .before = 1)


median_effects_df %>%
  kbl(booktabs = T) %>%
   kable_styling(latex_options = c("striped","HOLD_position","scale_down"))
```

Effect	b_Treatment_Groupantibiotic	.lower	.upper	.width	.point	.interval
Total	0.7322853	0.5651788	0.9419276	0.95	median	qi
Direct	0.8102662	0.6172184	1.0730615	0.95	median	qi
Indirect	0.9039691	0.6150431	1.3081756	0.95	median	qi

```r
data.frame("draw" = 1:length(total$b_Treatment_Groupantibiotic),
           "Total" = exp(total$b_Treatment_Groupantibiotic),
           "Direct" = exp(direct$b_Treatment_Groupantibiotic),
           "Indirect" = exp(indirect$b_Treatment_Groupantibiotic)) %>%
  pivot_longer(-draw) %>%
  ggplot(aes(x = value, fill = name)) +
  geom_density(alpha = .5) +
  theme_minimal() +
  geom_vline(aes(xintercept = b_Treatment_Groupantibiotic),
             color = "darkgreen",data = median_effects_df) +
  geom_vline(aes(xintercept = b_Treatment_Groupantibiotic),
             color = "darkred", linetype = "dashed",
             data = mean_effects_df)
```

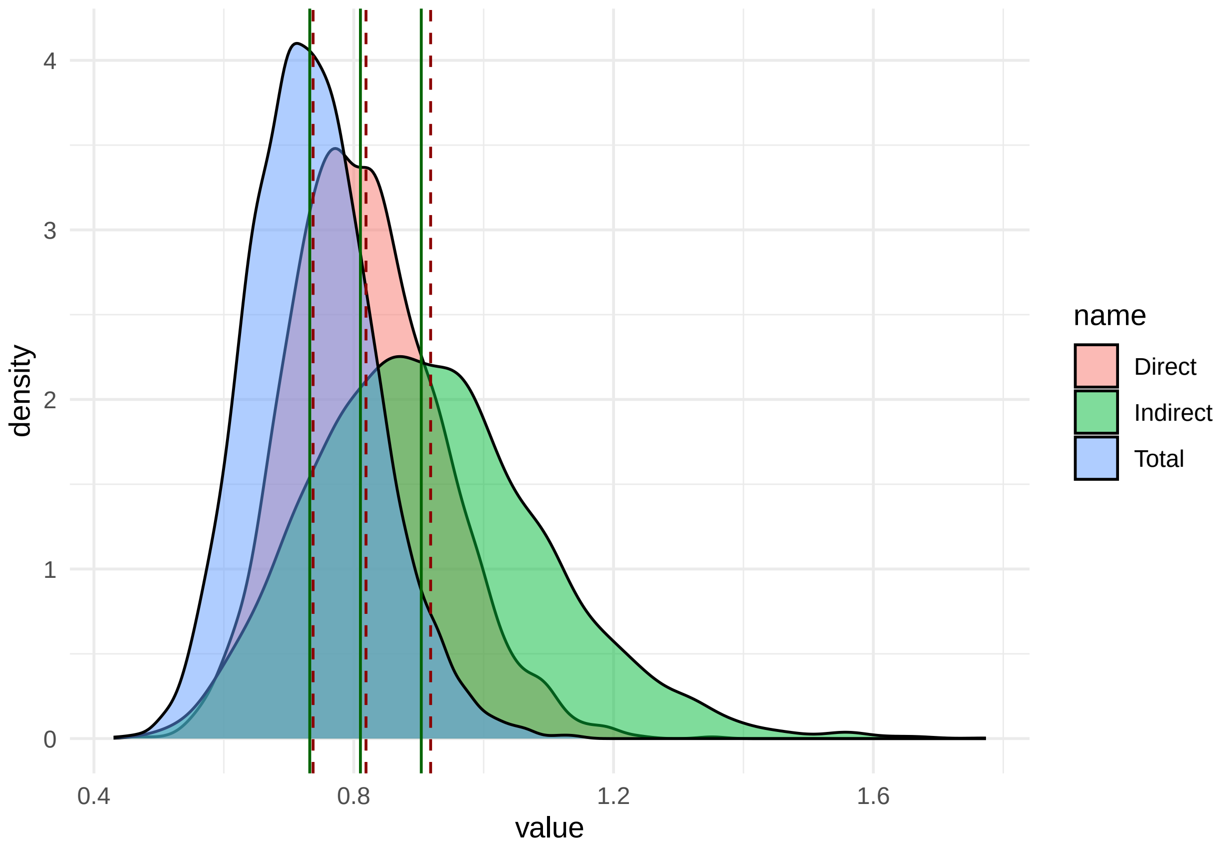


```
library(tidybayes)

newdata <- expand_grid(
  Treatment_Group = c("placebo","antibiotic"),
  pattern_ordered = unique(model_df$pattern_ordered),
  AGE = unique(model_df$AGE),
  SMOKING = unique(model_df$SMOKING),
  SEX = unique(model_df$SEX),
  Number_Sites = mean(model_df$Number_Sites,na.rm = T)
)

new_draws <- fit_direct %>%
  epred_draws(newdata = newdata)

myDraws <- new_draws %>%
  ungroup() %>%
  dplyr::select(Treatment_Group,SMOKING,AGE,pattern_ordered,.row,.draw,.epred) %>%
  drop_na() %>%
  group_by(Treatment_Group,SMOKING,AGE,pattern_ordered)


nm <- setNames(rep(c(1,4), c(2, 2)), c(rep(" ", 2), rev(unique(myDraws$Treatment_Group))))

myDraws %>%
  tidybayes::median_qi() %>%
  mutate(across(all_of(c(".epred",".lower",".upper")),round,2)) %>%
```

```r
dplyr::select(Treatment_Group,SMOKING,AGE,pattern_ordered,.epred) %>%
mutate(.epred = (.epred)/ mean(model_df$Number_Sites,na.rm = T)*100) %>%
pivot_wider(names_from = c(Treatment_Group,pattern_ordered),values_from = .epred) %>%
mutate(across(where(is.numeric),round,2)) %>%
setNames(sub(".*_","",names(.))) %>%
kbl(booktabs = T) %>%
 kable_styling(latex_options = c("striped","HOLD_position","scale_down")) %>%
add_header_above(nm)
```

		antibiotic				placebo			
SMOKING	AGE	non.responder	indifferent	short.term.responder	long.term.responder	non.responder	indifferent	short.term.responder	long.term.responder
Non-Smoker	<45	6.63	6.05	4.74	4.52	8.19	7.48	5.86	5.56
Non-Smoker	45<55	7.80	7.11	5.57	5.30	9.61	8.78	6.87	6.52
Non-Smoker	>55	9.67	8.82	6.92	6.57	11.95	10.85	8.52	8.13
Smoker	<45	12.26	11.11	8.72	8.35	15.13	13.75	10.79	10.27
Smoker	45<55	14.34	13.05	10.24	9.73	17.71	16.11	12.64	12.02
Smoker	>55	17.82	16.19	12.69	12.09	22.00	20.01	15.67	15.00
