# Supplementary figures and images for: The role of the oral microbiota in the causal effect of adjunctive antibiotics on clinical outcomes in stage III–IV periodontitis patients
Source: Microbiome. 2024 Oct 26;12:220. doi: 10.1186/s40168-024-01945-3 (PMC11515798; doi:10.1186/s40168-024-01945-3)

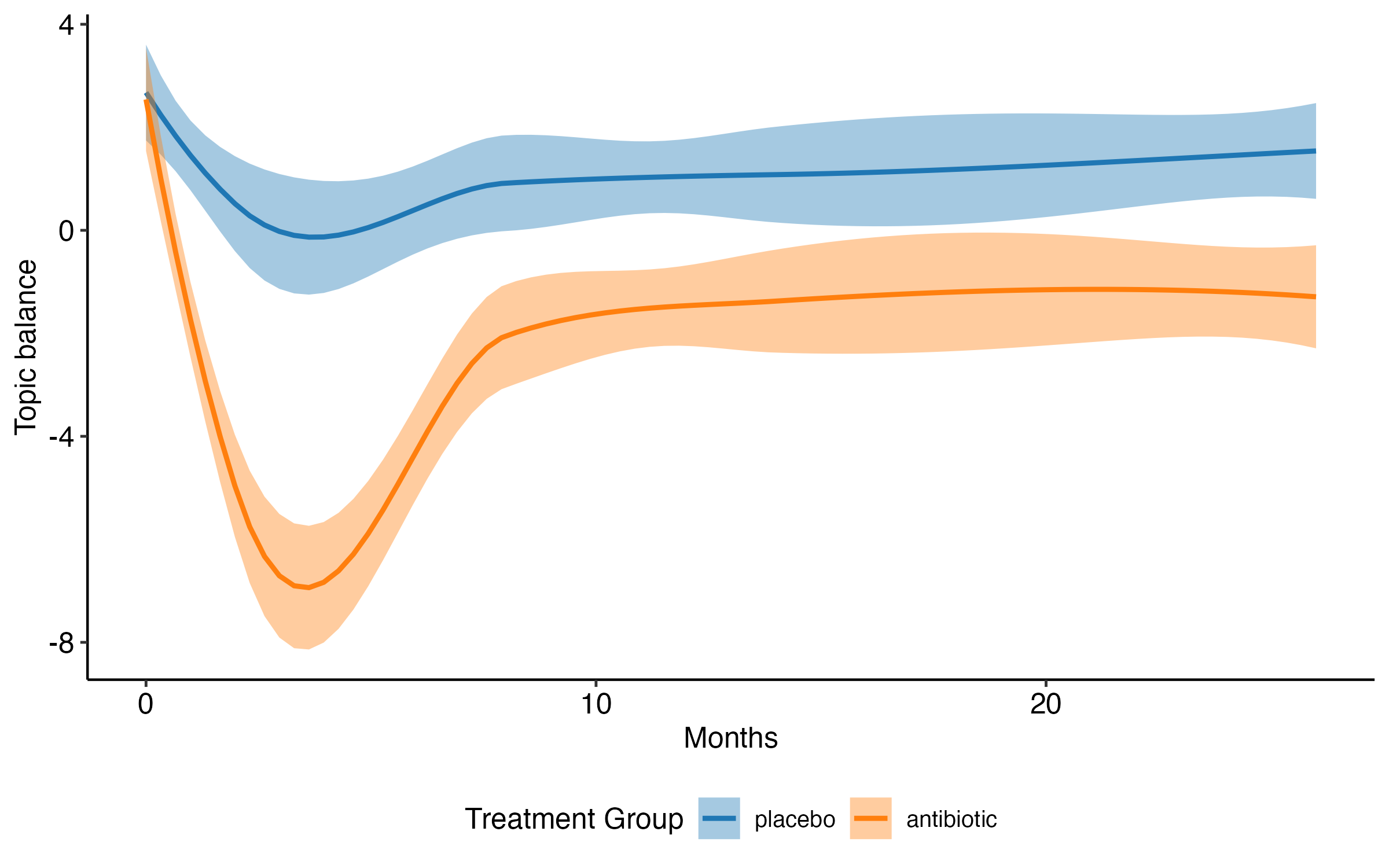

Supplement: Supplementary file 2 — Supplementary Material 1. [file 40168_2024_1945_MOESM1_ESM.zip › Supp_Figure1.tiff]
